# Clinical Applications of Artificial Intelligence in Periodontology: A Scoping Review

**DOI:** 10.3390/medicina61061066

**Published:** 2025-06-10

**Authors:** Georgios S. Chatzopoulos, Vasiliki P. Koidou, Lazaros Tsalikis, Eleftherios G. Kaklamanos

**Affiliations:** 1 Department of Preventive Dentistry, Periodontology and Implant Biology, School of Dentistry, Aristotle University of Thessaloniki, 54124 Thessaloniki, Greece; tsalikis@dent.auth.gr (L.T.); ekaklam@dent.auth.gr (E.G.K.); 2 Division of Periodontology, Department of Developmental and Surgical Sciences, School of Dentistry, University of Minnesota, Minneapolis, MN 55455, USA; 3 Centre for Oral Immunobiology and Regenerative Medicine, Centre for Oral Clinical Research, Institute of Dentistry, Queen Mary University London (QMUL), London E1 4NS, UK; v.koidou@qmul.ac.uk; 4 School of Dentistry, European University Cyprus, Nicosia 2404, Cyprus; 5 Hamdan bin Mohammed College of Dental Medicine, Mohammed bin Rashid University of Medicine and Health Sciences (MBRU), Dubai P.O. Box 505055, United Arab Emirates

**Keywords:** artificial intelligence, diagnosis, treatment planning, dental imaging, periodontology

## Abstract

*Background and Objectives*: This scoping review aimed to identify and synthesize current evidence on the clinical applications of artificial intelligence (AI) in periodontology, focusing on its potential to improve diagnosis, treatment planning, and patient care. *Materials and Methods*: A comprehensive literature search was conducted using electronic databases including PubMed-MEDLINE, Cochrane Central Register of Controlled Trials, Cochrane Database of Systematic Reviews, Scopus, and Web of Science™ Core Collection. Studies were included if they met predefined PICO criteria relating to AI applications in periodontology. Due to the heterogeneity of study designs, imaging modalities, and outcome measures, a scoping review approach was employed rather than a systematic review. *Results*: A total of 6394 articles were initially identified and screened. The review revealed a significant interest in utilizing AI, particularly convolutional neural networks (CNNs), for various periodontal applications. Studies demonstrated the potential of AI models to accurately detect and classify alveolar bone loss, intrabony defects, furcation involvements, gingivitis, dental biofilm, and calculus from dental radiographs and intraoral images. AI systems often achieved diagnostic accuracy, sensitivity, and specificity comparable to or exceeding that of dental professionals. Various CNN architectures and methodologies, including ensemble models and task-specific designs, showed promise in enhancing periodontal disease assessment and management. *Conclusions*: AI, especially deep learning techniques, holds considerable potential to revolutionize periodontology by improving the accuracy and efficiency of diagnostic and treatment planning processes. While challenges remain, including the need for further research with larger and more diverse datasets, the reviewed evidence supports the integration of AI technologies into dental practice to aid clinicians and ultimately improve patient outcomes.

## 1. Introduction

Periodontal disease is a common condition characterized by infection and inflammation of the gums and bone supporting teeth. Gingivitis is the early stage, marked by gum inflammation, which can progress to periodontitis, a more severe form causing bone loss and potential tooth loss. Global prevalence is high, affecting 20% to 50% of the population [1]. Findings from the 2019 Global Burden of Disease Study indicate that roughly 3.5 billion individuals across the globe were affected by oral health problems [2,3]. Among them, about 1 billion adults worldwide have severe periodontal disease, representing roughly 14% of the global adult population. This severe form can significantly diminish quality of life by affecting appearance, chewing ability, and self-esteem [4,5]. In the United States, approximately 47.2% of adults were reported to have mild to severe periodontitis in 2019. The economic burden is considerable, with an estimated USD 54 billion lost globally in 2010 due to reduced productivity caused by severe periodontal disease [6].

During its early development, periodontal disease may not cause any symptoms or may exhibit subtle signs that individuals might not even recognize. Without timely treatment, however, it can progress to significant bone deterioration, causing tooth mobility and eventually tooth loss [7]. Early detection by dental professionals is paramount in halting the progression of this condition. Given its potential for asymptomatic or indistinct initial manifestations that patients themselves may overlook, dentists and hygienists must be highly skilled in recognizing subtle clinical signs during routine examinations. This necessitates thorough visual inspections, careful periodontal probing, and potentially radiographic assessments to identify early inflammation or minimal bone loss that might otherwise go unnoticed. By prioritizing comprehensive evaluations and staying abreast of the latest diagnostic techniques, dental professionals can play a critical role in intervening before significant bone resorption occurs, thereby preventing tooth mobility and eventual tooth loss, and ultimately safeguarding patients’ oral health and quality of life. Implementing advanced screening systems that are easily accessible to the public could significantly increase the number of oral health problem assessments.

Artificial intelligence (AI) is revolutionizing dentistry by becoming an integral part of everyday clinical practice. Its impact is wide-ranging, significantly improving how dental professionals diagnose conditions using imaging, plan treatments tailored to individual patients, manage their practice, and streamline their workflow [8]. AI enhances the analysis of dental images like conventional X-rays and CT scans through advanced algorithms, allowing for more accurate identification of problems such as dental caries, vertical root fractures, apical lesions, salivary gland diseases, maxillary sinusitis, maxillofacial cysts, cervical lymph node metastasis, osteoporosis, neoplasms and alveolar bone loss [9]. In treatment planning, AI uses predictive data to create personalized treatment approaches and refine the creation of orthodontic appliances like clear aligners [10,11]. Moreover, AI offers dentists real-time support for making clinical decisions and provides thorough risk evaluations, ultimately leading to better patient results [12,13,14,15,16,17]. AI also plays a role in professional training through sophisticated simulations. Additionally, robot-assisted surgery holds promise for performing precise procedures, increasing accuracy and potentially shortening recovery times [18]. While challenges like system integration and data security exist, AI demonstrably increases both efficiency and accuracy within dental practices. Deep learning (DL) techniques, particularly convolutional neural networks (CNNs), have proven highly effective in analyzing images for diagnosis across various medical fields, experiencing rapid advancements in the last ten years [19,20,21,22]. AI models are increasingly recognized as promising tools that can assist dental professionals with a wide range of challenges. Research suggests that AI has the potential to be a powerful asset in improving patient care and significantly easing the burden on clinicians. AI applications in periodontology hold promise for improving diagnosis, treatment planning, and patient care. A scoping review is a preliminary assessment of the extent and nature of a research area. It systematically maps the available evidence, identifies key concepts, theories, and sources of evidence, and can clarify definitions and conceptual boundaries. This makes it particularly useful for informing future research, identifying gaps in the literature, and determining the feasibility of a full systematic review. This scoping review aims to map the existing evidence on the clinical applications of artificial intelligence in periodontology, focusing on the range and nature of AI use in diagnosis, risk assessment, treatment planning, and outcome prediction for patients with periodontal diseases, and to identify any gaps in the current research landscape regarding diagnostic accuracy and treatment efficacy. Due to the heterogeneity of study designs, imaging modalities, and outcome measures, a scoping review approach was employed rather than a systematic review. This methodology allowed for a broad overview and critical discussion of the available evidence, providing a valuable synthesis of a rapidly evolving field. We hypothesized that AI applications in periodontology will demonstrate superior performance compared to traditional methods in terms of diagnostic accuracy, treatment planning, and prediction of treatment outcomes, ultimately leading to improved patient care and clinical outcomes.

## 2. Materials and Methods

### 2.1. Protocol and Registration

**Figure 1:**
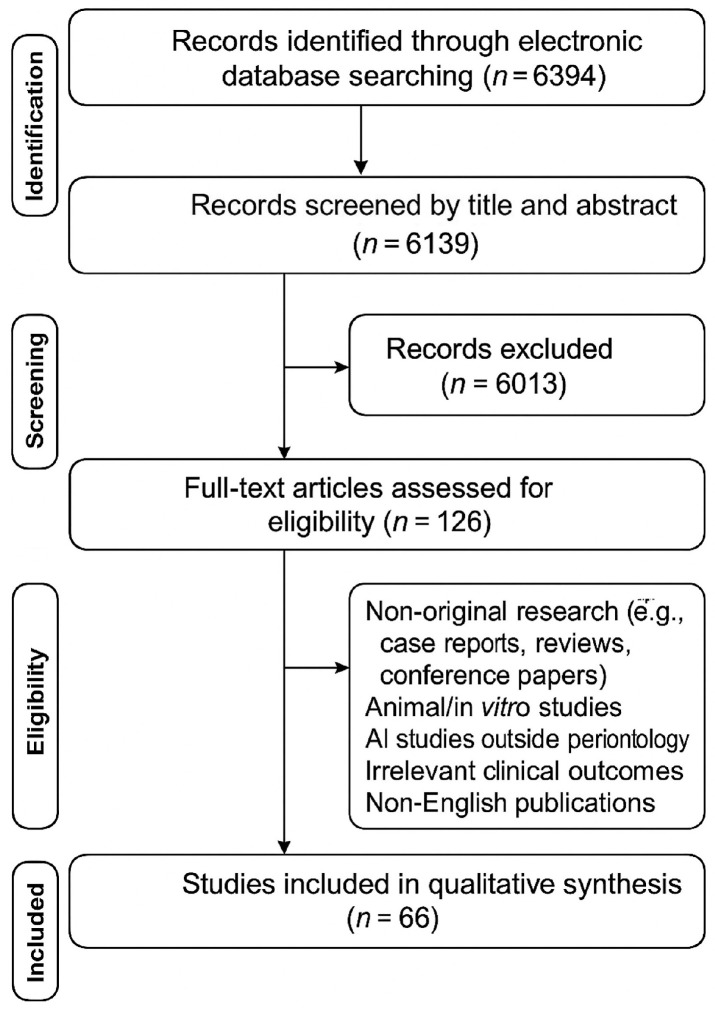
PRISMA flow diagram illustrating the study selection process.

The present review was registered online with the Open Science Framework (OSF) on 5 March 2025 under the following ID: osf-registrations-2m7wt-v1. This scoping review followed the methodological framework established by Peters et al. which is an update of the Joanna Briggs Institute methodology [23], with reporting guided by the PRISMA-ScR checklist [24]. The PRISMA flow diagram illustrating the study selection process is shown in Figure 1.

### 2.2. Eligibility Criteria

This review synthesized existing literature to address the central research question: What are the clinical applications of artificial intelligence in periodontology, and what is the evidence for their effectiveness and impact on patient outcomes? The research question for this review was structured using the PICO framework as follows:

- Population: Patients with periodontal diseases.
- Intervention: Artificial intelligence applications (e.g., diagnosis, risk assessment, treatment planning, outcome prediction).
- Comparison: Traditional methods or other AI applications.
- Outcome: Diagnostic accuracy, treatment efficacy.

The eligibility of studies for inclusion in this review was determined by the following inclusion and exclusion criteria:

#### 2.2.1. Inclusion Criteria

- Study Design: Clinical trials, cohort studies, case-control studies, cross-sectional studies.
- Population: Patients with any type of periodontal disease (gingivitis, periodontitis).
- Intervention: Any AI application used for diagnosis, risk assessment, treatment planning, or outcome prediction in periodontology.
- Outcomes: Diagnostic accuracy, treatment efficacy, patient-reported outcomes.
- Language: English.

#### 2.2.2. Exclusion Criteria

- Study Design: Case reports, case series, narrative reviews and systematic reviews, editorials, letters to the editor, conference papers/presentations.
- Population: Animal studies, in vitro studies.
- Intervention: AI applications not directly related to periodontology.
- Outcomes: Not relevant to clinical practice or patient care.
- Language: Non-English.

### 2.3. Information Sources and Search

A comprehensive literature search was conducted using electronic databases including PubMed-MEDLINE, Cochrane Central Register of Controlled Trials, Cochrane Database of Systematic Reviews, Scopus, Web of Science™ Core Collection, ProQuest Dissertations and Theses Global for studies published up to 3 February 2025. This review was conducted as a scoping review rather than a systematic review to accommodate the diverse range of topics addressed.

A manual review of the reference lists from the included articles was performed to identify additional relevant studies. There were no restrictions on the publication date. Two authors (G.S.C., V.P.K.) independently performed the database searches. Any discrepancies were resolved through discussion with a third author (E.K.) until consensus was achieved. The search strategy involved a combination of keywords based on the PICO framework. Details of the search strategy are presented in Table 1.

### 2.4. Selection of Sources of Evidence

The search results were initially checked for duplicates. Then, we assessed the eligibility of the remaining studies by examining their titles and abstracts. Relevant articles that passed this initial screening were retrieved in full text for detailed eligibility evaluation. Finally, studies that satisfied our PICO-based inclusion criteria were subjected to data extraction.

### 2.5. Data Charting Process and Data Items

Data were extracted using a pre-defined data extraction form. The extracted information included key study characteristics such as author(s), publication year, study location, population and size, study aims, and design. Furthermore, the extracted information encompassed data specific to the research question including details of AI model employed (type of algorithm, input data, training data, performance metrics) and outcome data including diagnostic accuracy (sensitivity, specificity, area under the curve-AUC) and other relevant findings and considerations.

### 2.6. Synthesis of Results

The included studies were categorized based on their primary AI application within periodontology, encompassing areas such as radiographic assessment of alveolar bone loss, the detection of intrabony and furcation defects using deep learning, automated gingivitis diagnosis, the automated identification of biofilm, calculus, and gingival inflammation, the application of deep and machine learning for overall periodontal disease detection and staging, the use of AI for enhanced dental diagnostics and patient communication, and other miscellaneous applications. Subsequently, a table was utilized to present a clear overview of the key AI methodologies and models employed across these categories, alongside their corresponding main findings and outcomes. Diagnostic accuracy outcomes, including sensitivity, specificity, and likelihood ratios, were summarized.

## 3. Results

### 3.1. Selection of Sources of Evidence

A total of 6394 records were identified through the electronic search. After removal of duplicates (n = 255), 6139 records remained for abstract screening. Upon exclusion of 6013 articles based on their abstracts, 126 articles remained for full-text evaluation. Following the exclusion of 60 articles based on full-text analysis, 66 articles remained for the inclusion in the review. Study designs other than original research articles, such as case reports, conventional reviews and systematic reviews, conference papers, investigations involving animals and in vitro studies were excluded. In addition, studies focusing on AI applications outside the direct scope of periodontology or reporting outcomes not relevant to clinical practice and patient care were also not considered. Finally, studies published in languages other than English were not included in this review.

#### Characteristics and Results of Sources of Evidence

A summary of the AI applications in periodontology is shown in Table 2. This table summarizes the applications of AI in periodontology, categorizing them into seven topics. Each category highlights the specific AI application focus, the key AI methodologies and models used, and the main findings or outcomes. The AI applications range from radiographic assessment using CNNs to detect bone loss, to employing deep learning for detecting bone defects and gingivitis, and for automated detection of biofilm and calculus. AI is also used for periodontitis detection and staging, enhancing dental diagnostics, and other diverse applications like predicting tooth extraction. Overall, the table demonstrates the broad utility of AI in periodontology, with AI models showing potential for accurate detection, classification, and prediction of various periodontal conditions.

### 3.2. Synthesis of Results

#### 3.2.1. Deep Learning Models for Radiographic Assessment of Alveolar Bone Loss

Accurate assessment of alveolar bone loss from radiographs is crucial for diagnosing and managing periodontal disease, but traditional methods can be time-consuming and subjective. To address these challenges, researchers have explored the use of AI, especially convolutional neural networks (CNNs), to automate and improve the analysis of dental radiographs. This section summarizes studies investigating the application of AI in radiographic bone loss assessment, highlighting both advancements and remaining limitations.

A study by Alotaibi et al. explored the use of a CNN named VGG16 to detect alveolar bone loss from periapical radiographs [25]. The researchers aimed to develop a computer-assisted detection system and evaluate its accuracy in identifying and categorizing the severity of bone loss due to periodontal disease. The CNN model was trained and tested on a dataset of 1724 periapical images. The results indicated that the CNN algorithm was useful in detecting alveolar bone loss, with a diagnostic accuracy of 73.0% for classifying normal versus disease and 59% for classifying the severity of bone loss. Another study introduced a web-based AI software, DiagnoCat, designed to detect periodontal bone loss in panoramic radiographs [26]. The AI software used two separate models: one for tooth detection, segmentation, and numbering, and the other for periodontal bone loss prediction. The tooth detection model was developed with Mask R-CNN, using ResNet101, while the bone loss prediction model was based on Cascade R-CNN architecture. The study’s findings suggest that DiagnoCat can effectively detect periodontal bone loss on panoramic radiographs. The AI performance, when compared to the consensus of clinicians, showed high F score, accuracy, and Cohen’s kappa coefficients for both tooth conditions and bone loss detection in binary and multi-class results. A study that utilized panoramic radiographs evaluated a total of 2276 images including 1137 bone loss cases and 1139 periodontally healthy cases [27]. A pretrained GoogLeNet InceptionV3 CNN was used, and the datasets were trained using transfer learning. The CNN system detected 99 of 105 cases with bone loss, with sensitivity, specificity, precision, accuracy, and F1 score of 0.94, 0.88, 0.89, 0.91, and 0.91, respectively. The study concluded that the CNN system can successfully determine periodontal bone loss and may help oral physicians with diagnosis and treatment planning in the future. A study that developed a machine learning model to automate the measurement of periodontal bone loss in panoramic radiographs and compared its performance to dentists consisted of three components: statistical inference to find probability functions, CNN to extract visual information, and an algorithm to calculate periodontal bone loss percentage and stage [28]. The model was tested against two radiologists, two periodontists, and one general dentist. The results showed that the model had acceptable performance for diagnosing slight to moderate bone loss but struggled with severe bone loss.

The preparation of dental panoramic radiographs and the deep learning approach for detecting and classifying periodontal bone loss were reported in a study by Chang et al. [29]. Radiographs were collected, anonymized, and divided into training, validation, and testing sets, with data augmentation. Periodontal bone level and cementoenamel junction level were annotated, and a modified Mask R-CNN model was used for segmentation. The classification process involved determining the principal axis of teeth/implants, calculating radiographic bone loss percentage, and staging periodontitis. Detection accuracy was assessed using the Jaccard index, pixel accuracy, and dice coefficient and the results showed high accuracy. For classification of periodontal bone loss, the automatic method showed minimal differences from radiologists’ diagnoses and correlation analysis confirmed a strong agreement between the CNN and radiologists. Similar results were reported when a dataset of 236 patients’ full-mouth radiographs was analyzed, with each tooth categorized by three periodontists [30]. Pre-processing and data augmentation were applied before using a multitasking InceptionV3 model and it achieved an average accuracy of 0.87 ± 0.01 in distinguishing mild (<15%) and severe (≥15%) bone loss, with sensitivity and specificity around 0.86–0.88. Using deidentified data from 270 patients, a deep learning ensemble model based on CNN was trained on 8000 periapical radiographs containing 27,964 teeth aiming to predict tooth position, detect shape, interproximal bone level, and radiographic bone loss [31]. The AI system, incorporating YOLOv5, VGG16, and U-Net architectures, was compared to clinicians’ assessments. The results showed high accuracy, with 92.61% for periodontal bone level detection, and 97.0% for RBL detection—outperforming dentists’ accuracy (76–78%).

A deep learning model integrating a CNN with a classification algorithm to enhance the efficiency and accuracy of periodontitis diagnosis for dentists was developed using periapical radiographs and clinical data [32]. Periapical radiographs and CNN models such as AlexNet and random forest classifications demonstrated strong diagnostic performance and may support dental professionals efficiently in identifying periodontitis stages. A deep learning system with an hourglass architecture was developed to automatically locate dental landmarks in X-rays, enabling the estimation of periodontal bone loss and disease severity for single, double, and triple-rooted teeth [33]. The model, enhanced with a modified data augmentation technique, achieved strong landmark detection accuracy (83.3%) and reasonably accurate periodontal bone level estimation (10.69% average error) and disease staging (58% accuracy, comparable to clinician variability). Vision transformer networks (ViT-base/ViT-large from Google, BEiT-base/BEiT-large from Microsoft, DeiT-base from Facebook/Meta) may increase the diagnostic performance and support the clinical use of such AI-based models [34].

A study that aimed to evaluate the accuracy of deep learning for classifying periodontal bone loss stages (healthy, Stage 1/2, Stage 3/4) using panoramic radiographs used three pre-trained CNN models (ResNet50, DenseNet121, InceptionV3) [35]. A dataset of 2533 panoramic radiographs was used showing that a new model combining DenseNet121 features extracted using global average pooling (GAP), followed by the minimum redundancy maximum relevance (mRMR) for feature reduction, and classified with a support vector machine achieved the highest performance in classifying periodontal bone loss. This method effectively identified relevant features from the images without requiring manual feature selection, demonstrating its potential for improved diagnostic accuracy compared to existing methods. Another study investigated the potential of five different CNNs (ResNet18, MobileNetV2, ConvNeXT/small, ConvNeXT/base, and ConvNeXT/large) to automatically detect periodontal bone loss on dental radiographs [36]. Researchers trained the CNNs using a large dataset of anonymized periapical radiographs categorized by dentists into no, mild, moderate, or severe periodontal bone loss. The performance of the CNNs was similar, with overall accuracy ranging from 82.0% to 84.8%, high sensitivity (88.8-90.7%), moderate specificity (66.2-71.2%), and a strong ability to discriminate between classes (AUC 0.884-0.913). However, the accuracy varied depending on the location in the mouth, with the best results for the mandibular anterior teeth and the poorest for the maxillary posterior teeth. The study concluded that while automatic PBL assessment is feasible, the diagnostic accuracy is location-dependent, highlighting the need for future research to enhance performance across all tooth groups.

A two-stage deep learning model combining U-Net and YOLOv4 to first locate teeth and key points and then calculate the percentage of periodontal bone loss for staging periodontitis was reported by Jiang et al. [37]. The model achieved an overall classification accuracy of 0.77, with varying performance across different tooth positions and severity levels. Importantly, the AI model generally outperformed general dental practitioners in classifying periodontal bone loss. An AI model using YOLOv8 to automatically assess periodontal bone loss and predict tooth prognoses from panoramic X-rays achieved high accuracy in segmenting teeth (97%) and in identifying key landmarks like the CEJ and bone level (98%) [38]. This AI-driven approach offers a faster and more accurate alternative to manual methods for periodontal diagnosis and prognosis. Another deep learning method (DeNTNet) that utilizes CNNs was tested for the detection of periodontal bone loss using panoramic dental radiographs [39]. Trained and validated on 11,379 annotated radiographs and tested on 800, DeNTNet achieved an F1 score of 0.75 on the test set, outperforming the average F1 score of dental clinicians, which was 0.69. The study highlighted the potential of DeNTNet as an automated diagnostic support system for periodontal bone loss. Another two-stage periodontitis detection convolutional neural network (PDCNN) was designed to improve the accuracy and implementation of automated image analysis for periodontal disease utilizing an anchor-free encoding for faster and more accurate predictions [40]. According to the findings, PDCNN achieved a radiographic bone loss classification accuracy of 0.762, outperforming state-of-the-art detectors like Faster R-CNN and YOLOv4. The impact of various image resolution improvement methods including traditional interpolation techniques and deep learning-based Super-Resolution CNN (SRCNN) as well as Super-Resolution GAN (SRGAN) on periodontal disease assessment in oral imaging was examined [41]. The findings revealed that while deep learning methods significantly improved the visual quality of the low-resolution images, this improvement did not consistently translate to better performance in the CNN classifiers for periodontal disease assessment.

**Figure 2:**
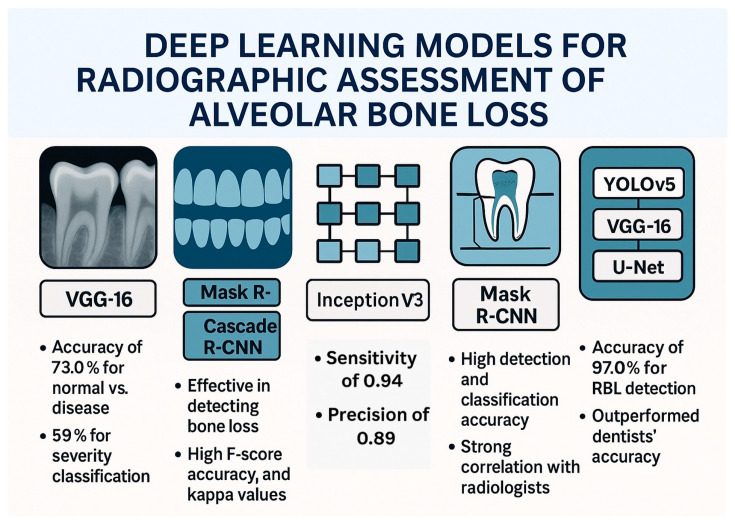
Deep learning models for radiographic assessment of alveolar bone loss.

In summary, a multitude of studies have explored the application of deep learning, predominantly using CNNs to automate the detection and classification of periodontal bone loss from both periapical and panoramic dental radiographs. These investigations have showcased the potential of various CNN architectures and methodologies to achieve high accuracy in identifying bone loss, categorizing its severity, and even predicting tooth prognoses, often demonstrating performance comparable to or even surpassing that of dental professionals. While challenges remain, such as variations in accuracy across tooth locations and difficulties with severe cases, the collective findings strongly suggest that AI-powered systems hold significant promise as valuable tools for enhancing the efficiency and accuracy of periodontal disease diagnosis and treatment planning in dentistry. Figure 2 demonstrates the deep learning models for radiographic assessment of alveolar bone loss.

#### 3.2.2. Deep Learning Approaches to Detect Intrabony and Furcation Defects in Periodontal Disease

Studies have evaluated the efficacy of models ranging from traditional machine learning algorithms to advanced CNNs and novel architectures like the Vision Transformer (ViT) in detecting and classifying a spectrum of periodontal bone loss patterns, including intrabony defects and furcation involvements, across different radiographic modalities. A study introduced an innovative approach using deep learning and image processing to help dentists assess periodontal disease by analyzing radiographic defect angles in intrabony defects [42]. The study classified intrabony defects as severe (greater than 37 degrees) or mild (less than 37 degrees) based on the radiographic defect angle and employed image enhancement techniques to improve diagnostic accuracy of the subsequent CNN analysis which used the YOLOv8 model. The CNN then classified the severity of periodontal lesions based on the radiographic defect angle. Another study explored the use of machine learning to classify periodontal defects in 2D periapical images [43]. Researchers compared human observation against evaluations from a radiomics platform, using support vector machine analysis. While both human observers and the machine learning model differed from the “gold standard,” the latter model performed similarly to human observers in detecting defects. The study suggested that machine learning can predict periodontal defects by analyzing specific radiomic features and image variables, potentially aiding clinical practitioners and even replacing human evaluations in the future.

A retrospective study developed a deep learning algorithm to interpret panoramic radiographs and detect periodontal bone loss and bone loss patterns [44]. The study used 1121 panoramic radiographs. Bone loss in the maxilla and mandible, interdental bone loss, and furcation defects were labeled using the segmentation method. Interdental bone loss was further categorized into horizontal and vertical. A CNN-based AI system was developed using U-Net architecture, and its performance was evaluated using the confusion matrix and ROC curve analysis. The AI system demonstrated the highest diagnostic performance in detecting total alveolar bone losses (AUC = 0.951) and the lowest in detecting vertical bone losses (AUC = 0.733). The study concluded that AI systems show promise in identifying periodontal bone loss patterns and furcation defects from dental radiographs. The authors suggested that CNN algorithms could be used to provide more detailed information, like automatic determination of periodontal disease severity and treatment planning. Another radiographic-based study introduced a deep learning approach using CNN to detect furcation defects in periapical radiographs with a reported accuracy of 95% [45]. The research utilized a dataset of 300 periapical radiographs and employed image preprocessing and masking techniques to enhance the visibility of furcation defects. The proposed segmentation algorithm achieved an overall accuracy of 94.97%, outperforming conventional methods, and the CNN model demonstrated identification rates of furcation involvement ranging from 92.96% to 94.97%. The authors suggested that this AI-assisted approach has the potential to improve the accuracy and efficiency of dental diagnosis, leading to better periodontal diagnosis, treatment planning, and patient outcomes.

In addition, a study investigated the use of deep learning, specifically CNN models, to automatically classify three-wall intrabony defects on intraoral dental X-rays [46]. Researchers trained six different CNN models (InceptionV3, InceptionResNetV2, ResNet50V2, MobileNetV3Large, EfficientNetV2B1, and VGG19) using a dataset of 1369 radiographs from 556 patients who had undergone periodontal surgery. The radiographs were categorized based on the presence or absence of three-wall defects. The performance of the models was evaluated using various metrics, with an AUC of 0.7 or higher considered acceptable. The results showed that when excluding circumferential defects from bite-wing radiographs, several models achieved acceptable AUC values (0.70–0.77), with the VGG19 model demonstrating the best performance (accuracy: 0.75, precision: 0.78, recall: 0.82, specificity: 0.67, NPV: 0.88, F1 score: 0.75). The study concluded that CNN models have the potential for clinical application in periodontal examination, diagnosis, and treatment planning for periodontal surgery by assisting in the identification of three-wall intrabony defects on intraoral radiographs. Another study investigated the accuracy of a deep learning model (ResNet101V2) in detecting furcation involvements, using axial Cone-Beam Computed Tomography (CBCT) images [47]. The researchers started with a dataset of 285 CBCT images (143 normal, 142 with furcation involvement) and used data augmentation to expand the training dataset to 600 images. The model was then tested on a separate set of 85 images. The results showed high performance with a training accuracy of 98%, validation accuracy of 97%, and a test accuracy of 91%. The model also achieved a precision and F1 score of 0.98, and an AUC of 0.98. The test loss was 0.2170. The study concluded that the ResNet101V2 deep learning model can accurately detect furcation involvement in axial CBCT images. However, the authors noted that this was a preliminary study with a relatively small dataset and suggested that future research with a larger dataset is needed to further confirm the accuracy of deep learning models for this purpose.

Classification of furcation involvement in mandibular molars using periapical radiographs was attempted in another investigation [48]. Researchers screened full mouth X-ray series and selected diagnostic-quality periapical radiographs of mandibular premolars and molars, categorizing the molar images as either “healthy” or having “furcation defects.” These images were divided into training, validation, and testing datasets and preprocessed. A CNN model, ResNet18, was trained and refined using the PyTorch framework. The model’s performance was evaluated using various metrics, including accuracy, sensitivity, specificity, and the area under the ROC curve. The results showed that the trained ResNet18 algorithm achieved a high accuracy of 96.47% in classifying healthy versus furcation involved molars in the testing set. The study concluded that this deep learning algorithm showed significant promise as a supplementary tool for detecting and managing periodontal diseases by accurately identifying mandibular molar furcation involvement on periapical radiographs. Finally, a study compared the performance of a novel deep learning model, the Vision Transformer (ViT), with traditional deep learning models (MLP, VGGNet, GoogLeNet) in classifying molars with or without furcation involvement using panoramic radiographs [49]. The researchers used a database of 1568 tooth images from 506 panoramic radiographs to train and evaluate the models. The results showed that the ViT model outperformed all other models, achieving the highest precision (0.98), recall (0.92), F1 score (0.95), and accuracy (92%), along with the lowest cross-entropy loss (0.27) and the highest AUC of 98%. The superior performance of ViT was statistically significant (*p* < 0.05). Gradient-weighted class activation mapping (Grad-CAM) analysis highlighted the relevant image areas that the ViT model focused on for its predictions. The study concluded that deep learning algorithms, particularly the ViT model, can effectively and automatically classify furcation involvement using readily available panoramic radiographs, potentially reducing the need for higher-cost and higher-radiation CBCT scans while improving diagnostic accuracy.

**Figure 3:**
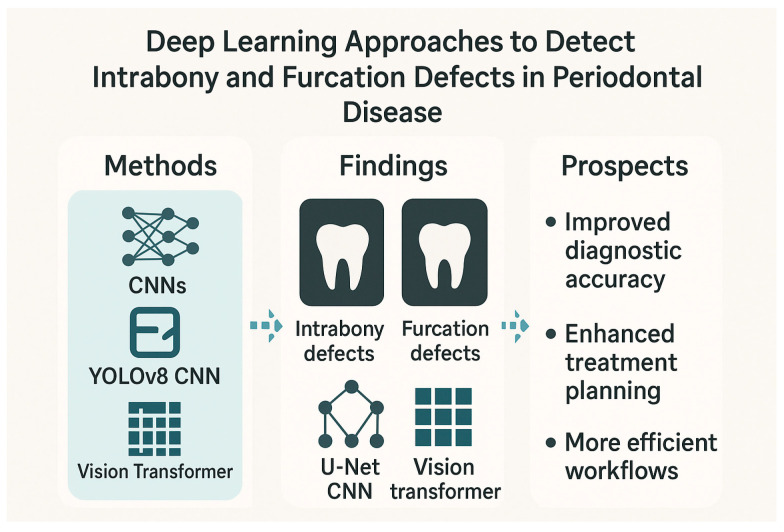
Deep learning approaches to detect intrabony and furcation defects in periodontal disease.

In conclusion, the current evidence strongly indicates the growing potential of AI, especially deep learning methodologies, to revolutionize periodontal disease diagnosis and assessment. By leveraging image processing and machine learning algorithms, these studies demonstrate the feasibility of automatically identifying and classifying various periodontal defects, including intrabony lesions and furcation involvements, across different radiographic modalities. While some studies highlighted the comparable performance of machine learning models to human observers, others showcased the superior accuracy and efficiency of advanced deep learning architectures like CNNs and the novel Vision Transformer. These AI-driven approaches offer the prospect of improved diagnostic accuracy, enhanced treatment planning, and potentially more efficient clinical workflows. However, the authors of several studies also acknowledge the need for further research with larger and more diverse datasets to validate these findings and facilitate their seamless integration into routine dental practice. As these technologies continue to evolve, AI-assisted tools are poised to become valuable assets for dental professionals in their efforts to combat periodontal disease and improve patient outcomes. Figure 3 shows the deep learning approaches to detect intrabony and furcation defects in periodontal disease.

#### 3.2.3. Automated Gingivitis Diagnosis

Gingivitis, a prevalent inflammatory condition of the gingiva, poses a significant threat to oral health and can progress to more severe periodontal diseases if left unaddressed. Traditional diagnostic methods often rely on subjective clinical assessments and manual record-keeping, which can be time-consuming and prone to variability. In response to these limitations, the application of AI, particularly deep learning techniques, has emerged as a promising avenue for enhancing the accuracy, efficiency, and accessibility of gingivitis detection and diagnosis. The following compilation summarizes several recent studies that explore diverse deep learning approaches, leveraging intraoral imaging and advanced computational models, to automatically identify, classify, and even grade gingival inflammation in various populations and clinical settings. These investigations highlight the potential of AI-driven solutions to revolutionize dental diagnostics, offering opportunities for early intervention, improved patient care, and broader public health impact.

A study aimed to develop and improve a classification model for diagnosing gingivitis by building a model using an artificial neural network and optimized it using fuzzy logic and multiple linear regression [50]. Fuzzy logic was used to create detection rules, while multiple linear regression was employed to measure analysis patterns and ensure optimal results. The study found that the optimization using fuzzy logic (generating 40 rules) and multiple linear regression (showing a significant correlation with an average value of 94.2%) produced excellent results. The researchers concluded that this optimized analysis model effectively enhances the gingivitis diagnosis process. Another study focused on using deep learning to automatically detect gingivitis in orthodontic patients through intraoral images [51]. Researchers developed two Faster R-CNN models using ResNet50. The first model accurately detected teeth (100% accuracy, precision, and mAP), identifying the region of interest. The second model detected gingival inflammation, achieving 77.12% accuracy, 88.02% precision, 41.75% recall, and 68.19% mAP. The study concluded that deep learning models show promise for detecting gingivitis in intraoral images, potentially aiding in early, non-invasive diagnosis and reducing the global impact of periodontal disease. A study presented a new automated method for diagnosing gingivitis using image processing and machine learning [52]. The method involves applying contrast-limited adaptive histogram equalization (CLAHE) to enhance the oral images, extracting features using the gray-level co-occurrence matrix (GLCM), and then classifying the images using an extreme learning machine (ELM). The researchers tested their method on a dataset of 93 images (58 gingivitis, 35 healthy) and reported an average sensitivity of 75%, specificity of 73%, precision of 74%, and accuracy of 74%. The study concluded that this new method is more accurate and sensitive compared to three other existing approaches for gingivitis diagnosis.

In addition, an investigation by Li et al. evaluated the effectiveness of several advanced deep convolutional neural network (ConvNet) models (AlexNet, VGG, GoogLeNet, ResNet) using ensemble learning to identify chronic gingivitis from oral screening images [53]. Researchers used a database of 683 intraoral images from 134 volunteers. The performance of the models was assessed by comparing their accuracy and sensitivity in recognizing gingivitis. The ResNet model achieved the highest AUC of 97%, followed by GoogLeNet (94%), AlexNet (92%), and VGG (89%). While ResNet and GoogLeNet performed best overall, there was no significant difference in sensitivity between ResNet, GoogLeNet, and AlexNet. However, the VGG sensitivity was significantly lower. The study concluded that ResNet and GoogLeNet show strong potential for efficiently diagnosing chronic gingivitis from images, which could aid doctors or even patients through self-examination. The same research group introduced a new Multi-Task Learning CNN model to screen for gingivitis, plaque accumulation, and calculus deposits from oral photos [54]. The goal was to provide a cost-effective and widely accessible solution for early detection, especially in areas with limited dental resources or for low-income populations, as routine dental visits can be unavailable or costly. The model was trained and evaluated on data from 625 patients, achieving classification AUC scores of 87.11% for gingivitis, 80.11% for dental calculus, and 78.57% for plaque accumulation. Importantly, the model could also localize these findings on the images with moderate accuracy, providing explainability to the screening results. Compared to general-purpose CNNs, the proposed Multi-Task Learning model showed significantly better performance in both classification and localization. It was ultimately shown that deep learning has strong potential for enabling widespread screening of dental diseases.

A deep learning network for automatically assessing the grade of gingival inflammation was developed by Wen et al. [55]. The researchers introduced a novel feature extraction method using T-distributed Stochastic Neighbor Embedding (t-SNE) for dimensionality reduction and built a CNN based on DenseNet for identifying and grading inflammation. To improve performance, they implemented a new teeth removal algorithm. They also used Grad-CAM++ to generate heatmaps for visualizing the model’s attention. The model achieved a mean intersection-over-union (MIoU) of 0.727 for gingivitis identification, and accuracy rates for five inflammation grades ranging from 73.68% to 79.22%. The area under the ROC values for the grades ranged from 0.80 to 0.84. The teeth removal algorithm significantly increased the model’s attention towards the gingival tissue and specifically the area near the gingival margin. The study concluded that the proposed deep learning model with the novel feature extraction method offers high accuracy and sensitivity for both identifying and grading gingival inflammation. Another study developed and evaluated a deep learning system called Oral-Mamba for segmenting intraoral photographic images to detect dental caries, dental calculus, and gingivitis, and to assess the severity of dental calculus [56]. Investigators collected 3365 intraoral images, which were labeled and divided into training, validation, and testing datasets. Oral-Mamba, a segmentation method based on Mamba architecture, demonstrated high accuracy in segmenting gingivitis (0.83), dental caries (0.83), and dental calculus (0.81). Notably, Oral-Mamba outperformed the U-Net model in IoU, accuracy, and recall, while also being 25% faster. Additionally, an intelligent evaluation model achieved 85% accuracy in classifying the degree of dental calculus. The authors demonstrated that this system offers a practical, intuitive, time-efficient, and cost-effective tool for assisting in the oral screening of dental caries, dental calculus, and gingivitis using intraoral camera images.

The use of AI to automatically provide visual plaque control advice by detecting gingivitis from intraoral photographs was explored in another investigation [57]. Researchers collected and labeled a dataset of intraoral frontal view images, categorizing gingival margin sites as healthy, diseased, or questionable. This data was split into training and validation sets and used to train a novel AI system. The AI performance in detecting gingivitis was then evaluated on the validation set, measuring sensitivity, specificity, and mean intersection-over-union (mIOU). The AI system demonstrated high sensitivity (0.92) and specificity (0.94) in correctly identifying healthy and diseased pixels. The mIOU of 0.60 was also above the acceptable threshold. The study concluded that AI can accurately identify specific sites with and without gingival inflammation, performing comparably to a visual examination by a dentist. This suggests that such a system could be valuable for monitoring the effectiveness of patients’ plaque control efforts. The same research team evaluated GumAI, a new smartphone-based AI tool for detecting gingivitis in older adults at community day-care centers [58]. Researchers compared the GumAI assessments of intraoral photos to those of a panel of dental professionals. GumAI showed high sensitivity (0.93), positive predictive value (0.90), accuracy (0.85), and F1 score (0.91), but lower specificity (0.50) and negative predictive value (0.56). Importantly, all participating older adults reported high acceptance of the tool and the personalized oral hygiene instructions it provided. The study showed that GumAI has strong potential for improving gingivitis detection and oral health management in community settings, despite needing further improvements in specificity and validation of usability measures. This research suggests that mHealth tools like GumAI could help expand oral healthcare access and reduce disparities.

Collectively, these studies underscore the significant strides being made in the application of deep learning for the automated detection and diagnosis of gingivitis. Utilizing a range of sophisticated architectures, including Faster R-CNN, Artificial Neural Networks optimized with fuzzy logic and MRL, novel CNNs with multi-task learning capabilities, and advanced segmentation models like Oral-Mamba and GC-U-Net, researchers have demonstrated the potential for achieving high levels of accuracy, sensitivity, and specificity in identifying gingival inflammation from intraoral images. Furthermore, the development of tools like GumAI for smartphone-based screening and systems capable of grading inflammation severity or localizing plaque and calculus highlights the versatility and clinical relevance of these AI-powered approaches. While some studies point to areas for further refinement, such as improving specificity or validating usability in broader contexts, the overall body of work strongly suggests that deep learning technologies hold considerable promise for transforming dental diagnostics, enabling more efficient, objective, and accessible methods for combating gingivitis and promoting oral health on a wider scale. Table 3 summarizes the AI models for automated gingivitis diagnosis.

#### 3.2.4. Automated Detection of Dental Biofilm, Calculus, and Gingival Inflammation Using Deep Learning

A number of studies have explored the capabilities of diverse AI models, including CNNs like U-Net and YOLO, as well as platforms like Google Cloud’s Vertex AI AutoML, in addressing critical aspects of dental health. These studies investigated the automated identification and quantification of dental biofilm (plaque), calculus, and gingival inflammation using various imaging modalities such as intraoral photographs, bite-wing X-rays, fluorescence imaging, and intraoral scans. By evaluating the performance of these AI-driven approaches against expert clinical assessments, these investigations collectively aim to demonstrate the potential of AI to enhance diagnostic accuracy, streamline clinical workflows, facilitate early detection, and ultimately improve patient outcomes in dental care.

A study by Andrade et al. investigated the ability of a U-Net neural network to automatically detect dental biofilm on tooth images. Researchers used two datasets of intraoral photographs [59]. The first dataset (96 photos with and without disclosing agents) validated the expert’s biofilm labeling. The second, larger dataset (480 photos with and without orthodontic appliances, without disclosing agents) was used to train the U-Net model to segment biofilm, with the dentist’s labels serving as the ground truth. The model’s performance was evaluated using accuracy, F1 score, sensitivity, and specificity. The U-Net model achieved an overall accuracy of 91.8%, F1 score of 60.6%, specificity of 94.4%, and sensitivity of 67.2%. Notably, the accuracy was slightly higher (92.6%) in images with orthodontic appliances. The study showed that using a U-Net for visually segmenting dental biofilm is a feasible approach that could potentially aid professionals and patients in identifying biofilm, thereby promoting better oral hygiene and health. CNN models for assessing dental plaque indices were validated in another study [60]. Researchers collected 210 intraoral images (frontal and lateral views) of plaque-disclosed teeth from 70 healthy adults. A three-stage method was employed: first, the YOLOv8 model detected teeth; second, the Segment Anything Model (SAM) segmented the detected teeth, creating a single-tooth dataset of 1400 images. Finally, a multi-class classification model called DeepPlaq was trained to index dental plaque based on the Quigley–Hein Index (QHI). The performance was evaluated using accuracy, recall, precision, and F1 score. The YOLOv8 teeth detector achieved a high accuracy (mAP of 0.941 ± 0.005). DeepPlaq demonstrated a maximum accuracy of 0.84 (probability of matching expert scores) and a small average scoring error of less than 0.25 on the QHI scale (0-5). The study concluded that the three-stage approach was excellent for detecting and segmenting teeth, and the DeepPlaq model showed strong potential for accurately assessing dental plaque indices.

The feasibility of using Google Cloud’s Vertex AI AutoML to automatically detect plaque levels on undyed photographs of permanent teeth was explored in another investigation, aiming to overcome the limitations of manual assessment and plaque-disclosing dyes [61]. Researchers collected undyed and corresponding dyed images of upper anterior teeth from 100 dental students. Plaque levels on dyed images were manually classified as mild (<30%), moderate (30–60%), or heavy (>60%) based on the stained surface area, serving as the ground truth for the undyed images. Two AutoML models were developed using the undyed images: a three-class model (mild, moderate, heavy) and a two-class model (acceptable vs. unacceptable). The models were evaluated using precision, recall, and F1 score. The three-class model achieved an average precision of 0.907, with the best performance in the heavy plaque category. The two-class model showed improved results with an average precision of 0.964 and an F1 score of 0.931. Overall, Vertex AI AutoML showed potential for non-invasive dental plaque detection, with the two-class model showing particular promise for clinical application. However, the authors recommended further research with larger datasets to improve the models’ generalization and real-world usability. Another study explored the ability of the advanced YOLO AI models (v9, v10, v11) to automatically identify three stages of dental plaque (new, mature, and over-mature) in order to improve early detection of plaque-related oral diseases [62]. Researchers collected 531 color images from 177 people using different smartphones after applying a disclosing gel. The images were then adjusted for consistent lighting and color. The YOLO models were trained to recognize the plaque stages, and their performance was evaluated. The YOLOv11m model performed best, especially in detecting older plaque. The study also found that newer plaque was harder to detect as it can look similar to gum tissue. The O’Leary index showed that most participants had significant plaque. In summary, the YOLO models showed promise for automatically detecting plaque in various real-world conditions, which could lead to better clinical efficiency, earlier diagnoses, and reduced oral health problems, particularly in areas with limited resources.

Wang et al. developed an automated, low-cost, and portable tool for early dental caries and calculus screening using fluorescence sub-band imaging and deep learning [63]. The method involved two steps: first, capturing six-channel fluorescence images of teeth by collecting imaging information under different fluorescence spectral bands. Second, a specialized 2D-3D hybrid convolutional neural network with an attention mechanism was used to classify and diagnose caries and calculus. Experimental results showed that this method performed competitively with existing approaches and this technology can be implemented on different smartphones, highlighting its potential for accurate, low-cost, and portable caries detection in community and home settings. The same research group used hyperspectral fluorescence imaging on 122 dental surfaces labeled by dentists, combined with machine learning algorithms [64]. The developed model fused features from spectra, textures, and colors using an integrated learning algorithm, resulting in high performance and strong generalization. The experimental results demonstrated that the diagnostic model achieved an accuracy of 98.6%, sensitivity of 98.4%, and specificity of 99.6% in identifying four different caries stages and calculus. The study concluded that this method can evaluate the entire tooth surface at the pixel level, offering enhanced discrimination and quantitative parameters, making it a promising new approach for early caries diagnosis. Another study aimed to create an automated method for objectively identifying gingival inflammation using intraoral scanning (IOS) and deep learning [65]. Researchers collected IOS images and periodontal probing data from 120 periodontitis patients. They used a deep learning model called GC-U-Net to automatically segment and identify inflamed gingival regions. The model achieved high accuracy, with a Dice coefficient of 77.8%, an IoU of 65.4%, and a pixel accuracy of 93.7%. Furthermore, the model’s identification performance showed a strong positive correlation with the sulcus bleeding index (SBI), a moderate positive correlation with the bleeding index (BI), and a negative correlation with probe depth (PD). Therefore, the authors displayed that this automated method provides a standardized and accurate auxiliary tool for clinical gingival inflammation examination, reducing subjective judgment and improving the reliability of diagnosis and treatment planning.

In pediatric dentistry, an AI model using a CNN was examined to automatically detect plaque on primary teeth [66]. The statistical analysis revealed no significant difference in diagnostic accuracy between the AI model and the pediatric dentist demonstrating clinically acceptable performance and suggesting its potential to aid in improving pediatric oral health. A similar study used images of 168 teeth from 20 patients, taken before and after plaque disclosing agent application [67]. Photos with disclosed plaque were used to train the AI to recognize non-disclosed plaque, with 140 teeth for training and 28 for testing. A dentist also reviewed the non-disclosed plaque images, and the AI performance was compared to the dentist’s using precision, sensitivity, F1 score, accuracy, specificity, AUC, and IoU. The AI system demonstrated higher performance with 82% precision, 84% sensitivity, 83% F1 score, 87% accuracy, 89% specificity, an AUC of 0.922, and an IoU of 76%. The dentist’s IoU was 0.71 and AUC was 0.833. The AI model’s performance was statistically significantly better than the dentist’s (*p* < 0.05). The study concluded that the developed AI algorithm shows promising and clinically acceptable results in detecting dental plaque compared to a dentist. Finally, a study introduced a new system for detecting dental calculus in bite-wing images using YOLOv8 for accurate tooth identification [68]. The researchers proposed a novel image-enhancement algorithm that combines a median and a bilateral filter to improve the accuracy of convolutional neural networks in classifying dental calculus by enhancing interdental edges. Before enhancement, the accuracy using GoogLeNet was 75.00%, which significantly increased to 96.11% after applying the enhancement algorithm. It was therefore demonstrated that this system has the potential to streamline dental consultations and improve the efficiency of dental services. Diagnocat AI software has also shown high sensitivity (above 0.8) for the detection of dental calculus on panoramic radiographs [69].

In summary, these studies provide compelling evidence for the growing utility of AI in the automated detection and assessment of key indicators of oral health, including dental biofilm, calculus, and gingival inflammation. Utilizing a range of deep learning architectures and imaging techniques, the research consistently demonstrates the potential for AI models to achieve high levels of accuracy, sensitivity, and specificity, often comparable to or even exceeding the performance of experienced dental professionals. From segmenting biofilm on tooth surfaces using U-Net to indexing plaque severity with DeepPlaq and identifying calculus in X-rays with enhanced YOLO models, these investigations highlight the versatility of AI in analyzing diverse dental imaging data. Furthermore, the exploration of accessible platforms like Vertex AI AutoML and the development of low-cost, portable tools based on fluorescence imaging suggest a future where AI-powered dental diagnostics can be more widely implemented, even in resource-limited settings. While acknowledging the need for further research with larger and more diverse datasets to ensure robust generalization and real-world applicability, the collective findings strongly support the integration of AI technologies into dental practice. This advancement promises to aid professionals in making more informed decisions, empower patients with a better understanding of their oral health, and ultimately contribute to improved preventative and therapeutic strategies for a range of common dental conditions. Table 4 summarizes the AI models for automated oral health detection.

#### 3.2.5. Deep Learning and Machine Learning for Periodontal Disease Detection and Staging

Studies have explored diverse approaches employing machine learning and deep learning techniques to analyze dental radiographic images and clinical data for the detection, staging, and prediction of periodontitis. These investigations utilize various imaging modalities, including panoramic and bite-wing radiographs, and employ a range of AI architectures, from convolutional neural networks and recurrent units to support vector machines and decision trees, all aimed at enhancing the accuracy and efficiency of periodontal disease assessment.

A study by Shon et al. developed a deep learning framework to automatically classify periodontitis stages in individual teeth using dental panoramic X-rays [70]. The model identified bone loss and the cementoenamel junction from patient data, and tooth number and length from the AIHub database. This integrated information was used to classify periodontitis into four stages based on the 2018 classification of periodontal diseases. Compared to dental specialists, the framework achieved a high accuracy (0.929), recall (0.807), and precision (0.724) and the authors claimed that this tool can aid dentists in diagnosis and treatment planning, with plans for a future application to support periodontal disease management. Similarly, Ertas et al. aimed to develop a machine learning-based decision system to simplify the staging and grading of periodontitis according to the 2018 classification [71]. Researchers used clinical data from 144 individuals to train various machine learning models, achieving high accuracy in staging with Decision Tree (97.2%), random forest, and k-nearest neighbor (98.6%) algorithms. Additionally, they explored using panoramic radiographic images processed with deep learning, with a hybrid ResNet50 and support vector machine model reaching 88.2% accuracy in staging. However, radiographic images were less successful in accurately modeling the grading of periodontitis. The study concluded that the developed decision system shows promise in aiding periodontal diagnoses, although further optimization is needed to improve results. Furthermore, another study explored the use of the YOLOv8 deep learning model to automatically stage periodontal bone loss based on bite-wing X-ray images [72]. A dataset of 1752 bite-wing images, classified into four stages of bone loss (healthy, mild, moderate, severe), was used to train and test the model using five-fold cross-validation. The model achieved a training accuracy of 86.10%, precision of 84.79%, recall of 82.35%, and F1 score of 84.41%. Therefore, it was concluded that the deep learning model showed successful results in staging periodontal bone loss in bite-wing images, with higher classification scores for healthy and severe cases due to their more distinct visual characteristics. Researchers trained and tested another deep CNN algorithm on a dataset of periapical radiographic images and showed that the diagnostic accuracy for periodontal compromised teeth was 81.0% for premolars and 76.7% for molars [73]. When predicting the extraction of clinically diagnosed severe periodontal destruction, the accuracy was 82.8% for premolars and 73.4% for molars.

In addition, a study developed a novel deep learning ensemble model based on CNNs to automatically analyze dental panoramic radiographs for tooth position detection, tooth outline and tissue segmentation, periodontal bone loss assessment, and periodontitis stage prediction [74]. The model, incorporating YOLOv8, Mask R-CNN, and TransUNet, was trained and evaluated on radiographs from 320 patients (8462 teeth). The deep learning method showed a periodontal bone loss degree deviation of 5.28% compared to expert annotations. The Pearson Correlation Coefficient between the deep learning method and periodontists’ diagnoses was 0.832 (*p* < 0.001), and the Intraclass Correlation Coefficient was 0.806 (*p* < 0.001). The overall diagnostic accuracy of the DL method was 89.45%. The study concluded that the proposed DL ensemble model demonstrates high accuracy and efficiency in radiographic detection, serving as a valuable tool for periodontal diagnosis, potentially enhancing clinical performance, preventing medical negligence, and acting as a learning resource for dental professionals. Similarly, 558 panoramic radiographs were cropped into 7359 individual tooth images and the model achieved an overall accuracy of 0.72, precision of 0.76, recall of 0.64, F1 score of 0.68, and a micro-average AUC of 0.79 in radiographic bone loss stage classification demonstrating reliability in assisting with the staging of radiographic bone loss [75]. Panoramic dental X-rays from 456 patients were utilized to evaluate the diagnostic accuracy of a radiographic-based periodontal bone loss method as a screening tool for periodontitis [76]. The results indicated that the method, when aligned with the 2018 case definition, is a reliable tool for screening periodontitis based on radiographic bone loss and the authors emphasized that it is a valuable screening tool that cannot replace comprehensive clinical evaluation. Researchers created a dataset of 238 panoramic images with annotations for teeth, alveolar bone contours, and cementoenamel junctions [77]. They employed a Mask R-CNN model for tooth segmentation and a U-Net model for segmenting alveolar bone and cementoenamel junctions. By analyzing the segmented teeth and bone structures and calculating the proportion of alveolar bone loss along the dental long axis (determined using the principal component analysis), the study evaluated its approach on 20 panoramic images (496 teeth) and achieved an accuracy rate of 90.73% in staging periodontitis.

A study by Vigil et al. proposed a new deep learning model called Adaptive DenseNet with Gated Recurrent Unit (AD-GRU), optimized by the Refined Red Kite Optimization Algorithm (RRKOA), to detect early periodontal bone loss from dental images [78]. The method involved segmenting teeth using DenseUNet++ and then feeding these segmented images to the optimized AD-GRU for bone loss detection, which is further used to determine the periodontitis stage. The proposed approach achieved a high accuracy of 94.45%, outperforming other models like LSTM, DenseNet, GRU, and DenseNet-GRU. An investigation by Ozden et al. focused on developing and comparing three machine learning algorithms—Support Vector Machine, Decision Tree, and Artificial Neural Networks—for the classification of periodontal diseases [79]. Using risk factors, periodontal measurements, and radiographic bone loss data from 150 patients, the researchers trained and tested these models to categorize individuals into six distinct periodontal conditions. The Decision Tree and Support Vector Machine algorithms demonstrated superior performance in disease classification, both achieving 98% accuracy, while the Artificial Neural Network showed significantly lower accuracy. The use of AI algorithms to diagnose periodontal disease from intraoral images of 60 patients with varying degrees of the condition was tested in another study [80]. The AI achieved an overall accuracy of 87%, with a sensitivity of 90% and specificity of 84%. These results were comparable to the clinical diagnoses made by experienced periodontal specialists (86% accuracy), with no statistically significant difference between the two methods. The study concluded that AI algorithms show promising potential for diagnosing periodontal disease using intraoral image analysis.

In summary, it has become quite obvious the considerable potential of AI-driven solutions in revolutionizing periodontal disease diagnosis and management. While each study employs unique methodologies and achieves varying degrees of success, the overarching trend indicates that machine learning and deep learning models can effectively analyze dental images and clinical data to detect bone loss, classify periodontitis stages, and even predict disease progression with promising accuracy, often comparable to or even exceeding the performance of human specialists. These advancements pave the way for the development of valuable computer-assisted tools that can aid dental professionals in early detection, treatment planning, and ultimately improving patient outcomes in the fight against periodontal diseases, although further research and optimization are often warranted for seamless clinical integration. Table 5 summarizes the AI applications in periodontal disease detection and staging.

#### 3.2.6. Harnessing Artificial Intelligence for Enhanced Dental Diagnostics and Patient Communication

Innovative applications of AI and machine learning in modern dentistry focus on enhancing diagnostic capabilities and patient communication. From automated gingival tissue analysis to comprehensive feature identification in intraoral images, these investigations highlight the transformative potential of AI in the dental field. A study investigated the use of CNNs in deep learning to automatically detect and measure keratinized gingiva in intraoral photographs [81]. Researchers compared the segmentation performance of different CNN architectures using 600 photographs. The ResNet50 model achieved the highest accuracy (91.4%) in identifying keratinized gingiva. Measurements of keratinized gingiva width taken by the ResNet50 model were in excellent agreement with measurements taken by clinicians, particularly when considering the bone and gingival phenotype. While there were some statistically significant differences in measurements based on who performed the measurement and the jaw being examined, the study concluded that the automated segmentation using the ResNet50 model is a promising and feasible method to assist dental professionals in evaluating keratinized gingiva, potentially saving time and reducing the need for extensive experience. A user-friendly software was created for dentists to visualize potential gingival recession in individual patients using 3D mouth models generated from intraoral scans of 1057 volunteers [82]. The software allows dentists to predict and simulate recession, including a slider for gradual demonstration, aiming to improve patients’ understanding and motivation for better oral hygiene. Another study developed and evaluated an AI model using the YOLOv5x architecture to automatically identify and segment various features in intraoral photographs [83]. These features included individual teeth with FDI numbering, frenulum attachments, gingival overgrowth areas, and signs of gingival inflammation. The model was trained on 654 labeled intraoral photographs, with a significant number of labels for each feature. The performance of the AI model was statistically evaluated using sensitivity, precision, F1 score, and AUC. The results showed high performance for tooth numbering (F1 score: 0.875, AUC: 0.989) and frenulum attachment detection (F1 score: 0.830, AUC: 0.827), with moderate performance for gingival overgrowth areas (F1 score: 0.714, AUC: 0.774) and gingival inflammation signs (F1 score: 0.777, AUC: 0.802). The study concluded that AI systems can effectively interpret intraoral photographs for automatic identification of anatomical structures and dental conditions, suggesting their potential to significantly advance digital dentistry in both clinical and academic settings.

Collectively, these studies demonstrate the significant strides being made in leveraging AI to improve the accuracy, efficiency, and patient-centeredness of dental practice. The promising results suggest a future where AI-powered tools are seamlessly integrated into clinical workflows, ultimately leading to better oral health outcomes.

#### 3.2.7. Other Applications

AI-powered solutions, such as image inpainting and super-resolution, automated diagnostic support for periodontal disease and tooth extraction, and risk assessment tools, hold considerable promise for improving clinical practice. A study addressed the challenge of limited field-of-view in bite-wing X-rays for deep learning algorithms predicting clinical attachment levels (CALs) [84]. The researchers developed an inpainting algorithm using generative adversarial networks (GANs) coupled with partial convolutions to predict the missing out-of-view anatomical information. Using a large dataset of bite-wing and periapical radiographs with corresponding clinician-recorded CAL, they trained and validated their model. The results demonstrated a statistically significant improvement in CAL prediction accuracy with the inpainting method (MAE of 1.04 mm) compared to methods without inpainting (MAE of 1.50 mm). The study concluded that using this GAN-based inpainting technique enhances the accuracy of CAL prediction from limited-view dental X-rays, bringing AI-based CAL assessment closer to the 1 mm clinical measurement standard. This suggests its potential as a valuable tool in assisting with periodontal disease diagnosis. A super-resolution algorithm using convolutional layers and ReLU activation was developed to enhance the quality of dental X-ray images for improved periodontal disease prediction [85]. The researchers trained the algorithm using 1500 dental X-ray images and evaluated its performance quantitatively using root mean square error (RMSE) and structural similarity (SSIM) to compare the enhanced images with higher-resolution originals. Additionally, they used no-reference image quality evaluators. The results demonstrated that the proposed super-resolution method significantly improved image similarity and no-reference quality scores by 1.86 and 2.14 times, respectively, compared to a standard bicubic upsampling technique. The study concluded that this super-resolution algorithm is effective for enhancing dental X-ray images and holds significant potential for improving early diagnosis and prediction accuracy of periodontal disease in various applications.

In addition, AI models may assist in the decision-making process for tooth extraction by providing an “extractability score” based on panoramic radiographs [86]. Researchers trained a ResNet50 deep learning network using 26,956 individual tooth images extracted from 1184 panoramic X-rays, classifying them as either “extraction-worthy” or “preservable.” The AI model’s performance was evaluated against dentists on a separate test dataset. The results showed that the best AI model achieved a significantly higher ROC-AUC of 0.901 in identifying preservable teeth compared to the average ROC-AUC of 0.797 for dentists. Similarly, the AI model outperformed dentists in Precision-Recall AUC. The study concluded that AI models can outperform dentists when predicting tooth extraction based solely on X-ray images, and that providing more contextual information to the AI improves its performance. The authors suggested that AI could be a valuable tool for monitoring at-risk teeth and reducing errors in extraction decisions. Another study investigated the use of an ANN to assess the grade (progression risk) of periodontitis in patients [87]. The ANN was trained using data from patients, including gender, age, smoking status, approximal plaque index, bleeding on probing, clinical attachment loss, and pocket depth. The ANN assessment of periodontitis grade showed no statistically significant difference compared to clinical periodontal assessments. The ANN demonstrated a sensitivity of 85.7% (correctly identified disease) and a specificity of 80.0% (correctly excluded disease). The overall accuracy of the ANN in correctly classifying patients according to their periodontitis grade was 84.2% in the training set, with 15.8% of patients being incorrectly classified. The authors concluded that ANNs have the potential to be a useful tool in dental practice for assessing periodontitis progression risk, but further research is necessary to validate these findings.

Deep learning models, particularly CNNs, may also automatically and accurately identify the cementoenamel junction (CEJ) in dental ultrasound images offering potential for chairside periodontal assessment and improved dental care efficiency [88]. A deep learning algorithm using Mask R-CNN has also successfully been created to automatically segment periodontal ligaments (PDLs) in CBCT images by defining them as the overlap between teeth and alveolar bone [89]. The model demonstrated high PDL segmentation accuracy across most tooth types in qualitative analysis and achieved good quantitative performance with an mIoU of 0.667 and mDSC of 0.799. This AI-driven method for automatic PDL segmentation in CBCT images holds promise for chair-side measurements, potentially enhancing the efficiency and accuracy of diagnosis and treatment planning in various dental specialties. Finally, AI-assisted dental monitoring can effectively remind patients to maintain oral hygiene at home, leading to improved periodontal health and long-term oral health quality of life, with the addition of health counseling providing further benefits [90].

## 4. Discussion

This scoping review aimed to synthesize the current body of literature concerning the application of AI in clinical periodontology. Our findings revealed a significant and rapidly growing interest in leveraging AI, particularly deep learning methodologies, to address various challenges in the diagnosis, treatment planning, and management of periodontal diseases. The reviewed studies explored a diverse range of clinical applications, including the detection and classification of alveolar bone loss, intrabony defects, furcation involvements, gingivitis, dental biofilm, and calculus. Furthermore, AI has been investigated for its potential in periodontal disease staging, risk assessment, prediction of tooth extractability, identification of anatomical landmarks, and even in enhancing patient communication and adherence to oral hygiene practices.

The studies extensively utilized CNN architectures for image analysis, demonstrating their effectiveness in extracting relevant features from dental radiographs and intraoral photographs. Various established CNN models, such as VGG16, ResNet (including ResNet18, ResNet50, ResNet101, ResNet50V2), Inception (including InceptionV3), DenseNet (including DenseNet121), MobileNet, EfficientNet, and YOLO (including YOLOv4, YOLOv5, YOLOv8, YOLOv11), were employed and often adapted or combined with other deep learning techniques like Mask R-CNN, U-Net, Recurrent Neural Networks (e.g., GRU), and even more recent architectures like Vision Transformers (ViTs). These models have shown promising results in tasks ranging from binary classification (e.g., healthy vs. diseased) to multi-class classification (e.g., different stages of periodontitis) and segmentation of periodontal structures. The reported diagnostic accuracies, sensitivities, and specificities often reached levels comparable to or even exceeding those of experienced dental professionals, highlighting the potential of AI to augment clinical decision-making.

Comparing the findings across studies focusing on alveolar bone loss detection reveals a trend towards higher performance with more advanced CNN architectures and the use of panoramic radiographs. While the earlier VGG16 model on periapical images achieved moderate accuracy [25], studies employing architectures like Mask R-CNN, Cascade R-CNN (in DiagnoCat), and pretrained networks like GoogLeNet InceptionV3 on panoramic images demonstrated superior results, often surpassing the diagnostic capabilities of clinicians [26,27]. This suggests that the wider field-of-view and potentially richer contextual information in panoramic radiographs, combined with the enhanced feature extraction of more sophisticated models and transfer learning, contribute to improved bone loss detection. Furthermore, ensemble models and those incorporating specific feature selection techniques, like the DenseNet121-mRMR-SVM combination, also showed promising outcomes [28]. However, some studies indicated limitations in accurately assessing severe bone loss and highlighted the dependency of accuracy on the tooth location, suggesting that further refinement is needed to ensure consistent performance across all clinical scenarios [28,36]. Interestingly, simply enhancing image resolution through super-resolution techniques did not guarantee improved performance in downstream classification tasks [34].

Comparing the findings for intrabony and furcation defect detection reveals that the choice of imaging modality and AI architecture significantly impacts performance. While YOLOv8 demonstrated utility in classifying intrabony defect severity based on radiographic angles, traditional machine learning with SVM on radiomic features achieved comparable results to human observers in general defect detection [42,43]. The U-Net architecture showed strong capability in detecting overall alveolar bone loss but struggled with the more specific tasks of identifying vertical defects and furcation involvements on panoramic radiographs [44]. In contrast, CNNs specifically designed for furcation defect detection on periapical radiographs achieved high accuracy [45]. When evaluating various CNNs for three-wall intrabony defects on intraoral radiographs, VGG19 emerged as the top performer [46]. Notably, ResNet architectures also demonstrated high accuracy in detecting furcation involvement, particularly ResNet101V2 on CBCT images and ResNet18 on periapical radiographs of mandibular molars [47,48]. Interestingly, the Vision Transformer (ViT) outperformed conventional CNNs for furcation involvement classification on panoramic radiographs, suggesting the potential of this newer architecture for specific periodontal tasks [49]. These findings highlight the importance of tailoring both the AI model and the imaging technique to the specific type of periodontal defect being investigated to achieve optimal diagnostic accuracy.

Studies on automated gingivitis detection reveal a variety of successful approaches utilizing different AI models and image analysis techniques. While an optimized artificial neural network combined with fuzzy logic and multiple linear regression demonstrated excellent potential, deep learning models, particularly CNNs, have shown robust performance in analyzing intraoral images [50,51,52]. Faster R-CNN achieved promising accuracy in gingival inflammation detection alongside perfect tooth detection, highlighting its object detection capabilities [50,51,52]. Comparative studies of various ConvNets indicated that ResNet and GoogLeNet architectures achieved the highest performance in chronic gingivitis identification based on AUC [53]. More recent architectures like DenseNet, coupled with novel feature extraction methods, and the Mamba-based Oral-Mamba model, which outperformed U-Net in segmentation tasks including gingivitis, showcase the continuous evolution and increasing sophistication of AI in this domain [55,56]. Finally, AI systems applied to intraoral photographs have demonstrated high sensitivity and specificity in detecting gingivitis, paving the way for accessible smartphone-based tools for self-assessment and remote monitoring, although some platforms may require further refinement to improve specificity [57,58].

Studies on the automated detection of biofilm, calculus, and gingival inflammation reveal the effectiveness of various AI approaches across different imaging modalities. U-Net architectures demonstrate feasibility for biofilm detection [59], while multi-stage methods incorporating YOLOv8 and SAM show promise for detailed plaque assessment [60]. AutoML platforms and advanced YOLO models also indicate potential for non-invasive plaque identification and staging [61]. Furthermore, specialized CNNs combined with advanced imaging techniques like fluorescence and hyperspectral imaging achieve very high accuracy in detecting both caries and calculus, and models like GC-U-Net excel in segmenting gingival inflammation from intraoral scans [63,64,65].

Studies on periodontal disease detection and staging demonstrate that deep learning frameworks, particularly those utilizing panoramic radiographs and advanced architectures like ensemble models and Adaptive DenseNets with GRUs, achieve high accuracy, often comparable to or even exceeding the performance of specialists [60,61,62,63,64,65,66,67,68,69,70,71,72,73,74,75,76,77,78]. While radiographic analysis with hybrid CNN-SVM models shows promise, machine learning models leveraging clinical data, such as decision trees and SVMs, can also achieve remarkably high accuracy in staging periodontal disease [70,71,72,73]. Furthermore, YOLOv8 shows success in staging bone loss on bite-wing images, and ANNs analyzing intraoral images offer diagnostic accuracy on par with experienced clinicians, highlighting the potential of diverse AI approaches across various data types and imaging modalities [74,75,76,77].

A systematic review by Macrì et al. (2024) explored the role and applications of AI in dental implant planning, highlighting its potential to enhance precision and efficiency [91]. The review, based on searches in PubMed and Scopus, indicated a growing interest in AI for implant planning, with evidence suggesting improvements in precision and predictability compared to traditional methods. Key AI applications identified include the automated detection of bones, maxillary sinus, neuronal structures, and teeth, signifying the latest advancements in the field. Despite these promising prospects for optimizing clinical outcomes and patient management, the authors also identified challenges such as the necessity for high-quality training data and a lack of standardization in protocols, concluding that further research is crucial to fully realize the AI potential and address implementation hurdles in clinical practice. A scoping review by Mohammad-Rahimi et al. (2022) examined the burgeoning application of deep learning in periodontology and oral implantology, identifying 47 relevant studies [92]. The review found that deep learning is being utilized for diverse tasks, including the detection of periodontal conditions and bone loss, classification of dental implant systems, and prediction of treatment outcomes, often demonstrating high performance. However, significant heterogeneity in study designs, inconsistent reporting, and a high risk of bias across most studies severely limit the comparability of findings and the overall robustness of the evidence, underscoring the need for more rigorous and standardized research in this promising field. While both reviews recognize the promising prospects of AI for improving clinical outcomes and patient management, they consistently emphasized critical limitations: the need for high-quality training data, a lack of standardized protocols, and the pervasive methodological weaknesses in existing research, all of which necessitate further rigorous investigation to fully harness the AI capabilities in clinical practice.

AI is rapidly transforming dentistry, as highlighted by two recent reviews that underscore its foundational principles and diverse applications [93,94]. A descriptive review emphasized the AI role in enhancing diagnostic accuracy through the analysis of radiographs and 3D scans, personalizing treatment plans, and streamlining workflows, with specific uses in detecting oral lesions, mapping caries, and classifying teeth. It also noted the AI utility in patient management via virtual assistants for consultations and scheduling, and in dental education through VR simulations. Furthermore, this review detailed the AI specialized applications across various dental fields, from pediatric dentistry to forensic odontology, while acknowledging challenges like implementation costs and data privacy. Complementing this, another comprehensive review traced the AI historical development, classified it into weak and strong AI, and detailed its widespread diagnostic applications, particularly with radiographic and optical images. This latter review also explored the synergy between Evidence-based Dentistry and Machine Learning, recommending the MI-CLAIM checklist for transparency, and ultimately summarized the AI critical contributions to dental diagnosis, decision-making, treatment planning, and outcome prediction.

### 4.1. Strengths and Limitations

This review benefits from its comprehensive approach, encompassing a wide spectrum of AI applications in periodontology and providing an overview of the various deep learning models and methodologies employed. By synthesizing the findings across numerous studies, we have identified key trends and promising areas of research. However, this review is not without limitations. The included studies exhibit heterogeneity in terms of datasets (size, source, imaging modality), methodologies, and evaluation metrics, which makes direct comparison challenging. Furthermore, the majority of the reviewed studies are retrospective and primarily focus on radiographic analysis. There is a relative paucity of research on the integration of AI with clinical examination data and its real-time application in clinical settings. The generalizability of the findings might also be limited by the specific populations and settings in which the studies were conducted. While a systematic review offers a rigorous approach to synthesizing evidence, the inherent heterogeneity in study designs, interventions, and outcome measures across the included literature necessitated a scoping review. The wide range of topics investigated within the application of AI in periodontology precluded a strict adherence to systematic review methodology and its associated quantitative synthesis. Therefore, this scoping review provides a broad overview and critical discussion of the available evidence.

### 4.2. Future Research

Future research should focus on addressing these limitations. Larger, multi-center studies utilizing diverse patient populations and incorporating both radiographic and clinical data are needed to validate the performance and generalizability of AI models. Prospective studies evaluating the impact of AI-powered tools on clinical workflow, treatment outcomes, and patient satisfaction would be invaluable. Further investigation into the interpretability and explainability of AI models in periodontology is crucial for building trust and facilitating their adoption by clinicians. Exploring the integration of AI into chairside diagnostic tools and patient education platforms also represents a promising direction. Finally, research focusing on the ethical considerations, data privacy, and regulatory aspects of implementing AI in periodontal practice is essential for responsible innovation in this field.

## 5. Conclusions

In conclusion, this review highlighted the prominent role of CNNs and their variants as the dominant AI architecture for analyzing dental images across various periodontology applications including enhanced diagnosis, treatment planning, and patient management. While panoramic radiographs are frequently utilized for broad assessments, periapical and bite-wing images remain crucial for detailed evaluations. The performance of AI models varies depending on the complexity of the task and the specific tooth location being analyzed, generally achieving higher accuracy in simpler detection tasks. Notably, AI often demonstrates performance comparable to or even exceeding that of clinicians in specific image analysis tasks. The development of ensemble models and task-specific architectures holds significant promise for further performance improvements, underscoring the critical importance of high-quality, accurately annotated training data for the success of all AI-driven approaches in this field.

## Figures and Tables

**Table 1:** Databases and keywords employed in the review.

Database	Search Strategy	Hits
PubMed-MEDLINE	((AI OR “Artificial Intelligence” OR “machine learning” OR “deep learning” OR “neural network” OR “convolutional network”)) AND (Periodontology OR periodontics OR periodontal disease OR periodontitis OR periodontium OR periodontal)	3195
Cochrane Central Register of Controlled Trials	((AI OR “Artificial Intelligence” OR “machine learning” OR “deep learning” OR “neural network” OR “convolutional network”)) AND (Periodontology OR periodontics OR periodontal disease OR periodontitis OR periodontium OR periodontal) in Title Abstract Keyword—(Word variations have been searched)	46
Cochrane Database of Systematic Reviews	((AI OR “Artificial Intelligence” OR “machine learning” OR “deep learning” OR “neural network” OR “convolutional network”)) AND (Periodontology OR periodontics OR periodontal disease OR periodontitis OR periodontium OR periodontal) in Title Abstract Keyword—(Word variations have been searched)	0
Scopus	TITLE-ABS-KEY((ai OR “Artificial Intelligence” OR “machine learning” OR “deep learning” OR “neural network” OR “convolutional network”)) AND (periodontology OR periodontics OR periodontal AND disease OR periodontitis OR periodontium OR periodontal)	3370
Web of Science™ Core Collection	((AI OR “Artificial Intelligence” OR “machine learning” OR “deep learning” OR “neural network” OR “convolutional network”)) AND (Periodontology OR periodontics OR periodontal disease OR periodontitis OR periodontium OR periodontal) (Topic) and Preprint Citation Index (Exclude—Database) Timespan: All years. Search language = Auto	824
ProQuest Dissertations and Theses Global	title(((AI OR “Artificial Intelligence” OR “machine learning” OR “deep learning” OR “neural network” OR “convolutional network”)) AND (Periodontology OR periodontics OR periodontal disease OR periodontitis OR periodontium OR periodontal)) OR abstract(((AI OR “Artificial Intelligence” OR “machine learning” OR “deep learning” OR “neural network” OR “convolutional network”)) AND (Periodontology OR periodontics OR periodontal disease OR periodontitis OR periodontium OR periodontal)) Filters activated: Full text	29

**Table 2:** Summary of artificial intelligence (AI) applications in periodontology.

Topic	AI Application Focus	Key AI Methodologies/Models	Key Findings/Outcomes
AI and Radiographic Assessment of Alveolar Bone Loss	Alveolar Bone Loss Detection and Classification	CNNs (VGG16, GoogLeNet InceptionV3), Mask R-CNN, Cascade R-CNN, YOLOv5, U-Net, Ensemble Models	- AI models show potential for accurate bone loss detection and classification. - Performance varies with CNN architecture, image type (panoramic vs. periapical), and task complexity. - Some models outperform clinicians in specific tasks.
Deep Learning for Intrabony and Furcation Defects	Detection and Classification of Bone Defects	YOLOv8, SVM, U-Net, CNNs (InceptionV3, ResNet), Vision Transformer (ViT)	- AI models can identify and classify intrabony defects and furcation involvements. - ViT shows promise for furcation involvement classification. - Choice of AI model and imaging modality is crucial.
Automated Gingivitis Diagnosis	Gingival Inflammation Detection and Grading	ANN, Faster R-CNN, ELM, ConvNets (ResNet, GoogLeNet), Multi-Task Learning CNN, DenseNet, Oral-Mamba	- AI models can accurately detect and grade gingivitis from intraoral images. - Multi-Task Learning CNNs can detect multiple conditions (gingivitis, plaque, calculus). - Smartphone-based tools show potential for accessible screening.
Automated Detection of Biofilm, Calculus, and Gingival Inflammation	Detection and Quantification of Dental Biofilm, Calculus, and Gingival Inflammation	U-Net, YOLO, SAM, DeepPlaq, AutoML, Hybrid CNNs, GC-U-Net	- AI models can effectively detect and quantify biofilm, calculus, and gingival inflammation. - Advanced imaging techniques (fluorescence, hyperspectral) enhance detection. - AI tools can aid in caries screening and gingival inflammation assessment.
Deep Learning and Machine Learning for Periodontal Disease Detection and Staging	Periodontitis Detection, Staging, and Prediction	Deep Learning Frameworks, Machine Learning (Decision Tree, SVM, KNN), YOLOv8, Ensemble Models, AD-GRU, ANN	- AI models can accurately classify periodontitis stages and predict disease progression. - Both radiographic data and clinical data are used. - Performance often comparable to or exceeding clinicians.
Harnessing AI for Enhanced Dental Diagnostics and Patient Communication	Automated Gum Tissue Analysis and Feature Identification	CNNs (ResNet50), YOLOv5x	- AI can accurately detect and measure keratinized gingiva. - AI can identify and segment various features in intraoral photographs.
Other Applications	Diverse Applications	GANs, Super-Resolution Algorithms, ResNet50, ANN, CNNs, Mask R-CNN	- AI can assist in CAL prediction, image enhancement, tooth extraction prediction, and periodontitis risk assessment. - AI can segment periodontal ligaments and aid in patient monitoring.

**Table 3:** AI models for automated gingivitis diagnosis.

AI Model/Approach	Primary Application	Performance
ANN with Fuzzy Logic	Enhanced diagnosis	94.2% correlation
Faster R-CNN	Detecting gingivitis in orthodontic patients	77.12% accuracy, 88.02% precision for inflammation
ELM (Image Processing)	Automated diagnosis	74% accuracy, 75% sensitivity
ResNet/GoogLeNet	Chronic gingivitis identification	ResNet AUC 97% (highest)
Multi-Task Learning CNN	Screening for gingivitis, plaque, calculus	Gingivitis AUC 87.11%
DenseNet CNN (Grading)	Assessing inflammation grade	73.68–79.22% accuracy for 5 grades
Oral-Mamba	Segmenting for caries, calculus, gingivitis	0.83 accuracy for gingivitis segmentation
AI System (Plaque Control)	Visual plaque control advice	0.92 sensitivity, 0.94 specificity
GumAI (Smartphone-based)	Gingivitis detection in community settings	0.85 accuracy, 0.93 sensitivity

**Table 4:** AI models for automated oral health detection.

Oral Health Indicator	AI Models/Methods Used	Key Findings/Impact
Dental Biofilm (Plaque)	U-Net, YOLOv8, DeepPlaq, Vertex AI AutoML, YOLOv9/v10/v11, CNNs	High accuracy in detection, segmentation, and indexing; potential for non-invasive and early detection.
Dental Calculus	Fluorescence imaging with 2D-3D hybrid CNN, YOLOv8, Diagnocat AI	High accuracy in detection across various imaging types (X-rays, fluorescence); enhances efficiency.
Gingival Inflammation	Faster R-CNN, ELM, ResNet, GoogLeNet, Multi-Task CNN, DenseNet CNN, Oral-Mamba, GumAI, GC-U-Net	High accuracy in detection, classification, grading, and localization; comparable to or exceeding human experts.

**Table 5:** AI application in periodontal disease detection and staging.

AI Model/Approach	Data Used	Key Application	Performance Highlights
Deep Learning Framework	Panoramic X-rays	Classify periodontitis stages (2018 classification)	92.9% accuracy, 80.7% recall, 72.4% precision
ML Algorithms (Decision Tree, Random Forest, k-NN, ResNet50 + SVM)	Clinical data, Panoramic X-rays	Simplify periodontitis staging/grading	Decision Tree and k-NN: 98.6% accuracy (staging clinical data); ResNet50 + SVM: 88.2% accuracy (staging radiographic)
YOLOv8	Bite-wing X-rays	Stage periodontal bone loss	86.10% accuracy, 84.79% precision, 82.35% recall
Deep CNN algorithm	Periapical radiographs	Diagnose periodontal compromised teeth; predict extraction	81.0% accuracy (premolars), 76.7% (molars) for diagnosis
Deep Learning Ensemble (YOLOv8, Mask R-CNN, TransUNet)	Panoramic radiographs	Tooth position, tissue segmentation, bone loss, periodontitis stage prediction	89.45% overall diagnostic accuracy; 0.832 Pearson Correlation with expert diagnosis
Mask R-CNN, U-Net	Panoramic images	Periodontitis staging (bone loss proportion)	90.73% accuracy in staging
Adaptive DenseNet with GRU (AD-GRU) optimized by RRKOA	Dental images	Detect early periodontal bone loss, determine periodontitis stage	94.45% accuracy
SVM, Decision Tree, ANN	Risk factors, periodontal measurements, radiographic bone loss	Classify periodontal diseases	SVM and Decision Tree: 98% accuracy
AI Algorithms (unspecified)	Intraoral images	Diagnose periodontal disease	87% accuracy, 90% sensitivity, 84% specificity (comparable to specialists)

## Data Availability

No new data were created or analyzed in this study.

## References

[1] WHO (2019). Global Status Report on Alcohol and Health 2018.

[2] Nazir M.A. (2017). Prevalence of periodontal disease, its association with systemic diseases and prevention. Int. J. Health Sci. (Qassim).

[3] Tichenor M., Sridhar D. (2019). Metric partnerships: Global burden of disease estimates within the World Bank, the World Health Organisation and the Institute for Health Metrics and Evaluation. Wellcome Open Res..

[4] Reynolds I., Duane B. (2018). Periodontal disease has an impact on patients’ quality of life. Evid. Based Dent..

[5] Tonetti M.S., Jepsen S., Jin L., Otomo-Corgel J. (2017). Impact of the global burden of periodontal diseases on health, nutrition and wellbeing of mankind: A call for global action. J. Clin. Periodontol..

[6] Listl S., Galloway J., Mossey P.A., Marcenes W. (2015). Global economic impact of dental diseases. J. Dent. Res..

[7] Könönen E., Gursoy M., Gursoy U.K. (2019). Periodontitis: A multifaceted disease of tooth-supporting tissues. J. Clin. Med..

[8] Rajpurkar P., Chen E., Banerjee O., Topol E.J. (2022). AI in health and medicine. Nat. Med..

[9] Khanagar S.B., Al-Ehaideb A., Maganur P.C., Vishwanathaiah S., Patil S., Baeshen H.A., Sarode S.C., Bhandi S. (2021). Developments, application, and performance of artificial intelligence in dentistry—A systematic review. J. Dent. Sci..

[10] Alam M.K., Kanwal B., Shqaidef A., Alswairki H.J., Alfawzan A.A., Alabdullatif A.I., Aalmunif A.N., Aljrewey S.H., Alothman T.A., Shrivastava D. (2023). A Systematic Review and Network Meta-Analysis on the Impact of Various Aligner Materials and Attachments on Orthodontic Tooth Movement. J. Funct. Biomater..

[11] Ruiz D.C., Mureșanu S., Du X., Elgarba B.M., Fontenele R.C., Jacobs R. (2025). Unveiling the role of artificial intelligence applied to clear aligner therapy: A scoping review. J. Dent..

[12] Alqutaibi A.Y., Algabri R., Ibrahim W.I., Alhajj M.N., Elawady D. (2024). Dental implant planning using artificial intelligence: A systematic review and meta-analysis. J. Prosthet. Dent..

[13] Koidou V.P., Chatzopoulos G.S., Tsalikis L., Kaklamanos E.G. (2025). Large Language Models in peri-implant disease: How well do they perform?. J. Prosthet. Dent..

[14] Dermata A., Arhakis A., Makrygiannakis M.A., Giannakopoulos K., Kaklamanos E.G. (2025). Evaluating the evidence-based potential of six large language models in paediatric dentistry: A comparative study on generative artificial intelligence. Eur. Arch. Paediatr. Dent..

[15] Chatzopoulos G.S., Koidou V.P., Tsalikis L., Kaklamanos E.G. (2024). Large language models in periodontology: Assessing their performance in clinically relevant questions. J. Prosthet. Dent..

[16] Giannakopoulos K., Kavadella A., Salim A.A., Stamatopoulos V., Kaklamanos E.G. (2023). Evaluation of the Performance of Generative AI Large Language Models ChatGPT, Google Bard, and Microsoft Bing Chat in Supporting Evidence-Based Dentistry: Comparative Mixed Methods Study. J. Med. Internet Res..

[17] Makrygiannakis M.A., Giannakopoulos K., Kaklamanos E.G. (2024). Evidence-based potential of generative artificial intelligence large language models in orthodontics: A comparative study of ChatGPT, Google Bard, and Microsoft Bing. Eur. J. Orthod..

[18] Jain S., Sayed M.E., Ibraheem W.I., Ageeli A.A., Gandhi S., Jokhadar H.F., AlResayes S.S., Alqarni H., Alshehri A.H., Huthan H.M. (2023). Accuracy Comparison between Robot-Assisted Dental Implant Placement and Static/Dynamic Computer-Assisted Implant Surgery: A Systematic Review and Meta-Analysis of In Vitro Studies. Medicina.

[19] Sharifi G., Hajibeygi R., Zamani S.A.M., Easa A.M., Bahrami A., Eshraghi R., Moafi M., Ebrahimi M.J., Fathi M., Mirjafari A. (2025). Diagnostic performance of neural network algorithms in skull fracture detection on CT scans: A systematic review and meta-analysis. Emerg. Radiol..

[20] Khadivi G., Akhtari A., Sharifi F., Zargarian N., Esmaeili S., Ahsaie M.G., Shahbazi S. (2025). Diagnostic accuracy of artificial intelligence models in detecting osteoporosis using dental images: A systematic review and meta-analysis. Osteoporos. Int..

[21] Rahman H., Khan A.R., Sadiq T., Farooqi A.H., Khan I.U., Lim W.H. (2023). A Systematic Literature Review of 3D Deep Learning Techniques in Computed Tomography Reconstruction. Tomography.

[22] Xiang B., Lu J., Yu J. (2024). Evaluating tooth segmentation accuracy and time efficiency in CBCT images using artificial intelligence: A systematic review and Meta-analysis. J. Dent..

[23] Peters M.D.J., Marnie C., Tricco A.C., Pollock D., Munn Z., Alexander L., McInerney P., Godfrey C.M., Khalil H. (2021). Updated methodological guidance for the conduct of scoping reviews. JBI Evid. Implement..

[24] Tricco A.C., Lillie E., Zarin W., O’Brien K., Colquhoun H., Levac D., Moher D., Peters M.D.J., Horsley T., Weeks L. (2018). PRISMA extension for scoping reviews (PRISMA-ScR): Checklist and explanation. Ann. Intern. Med..

[25] Alotaibi G., Awawdeh M., Farook F.F., Aljohani M., Aldhafiri R.M., Aldhoayan M. (2022). Artificial intelligence (AI) diagnostic tools: Utilizing a convolutional neural network (CNN) to assess periodontal bone level radiographically—A retrospective study. BMC Oral Health.

[26] Amasya H., Jaju P.P., Ezhov M., Gusarev M., Atakan C., Sanders A., Manulius D., Golitskya M., Shrivastava K., Singh A. (2023). Development and validation of an artificial intelligence software for periodontal bone loss in panoramic imaging. Int. J. Imaging Syst. Technol..

[27] Kurt Bayrakdar S., Çelik Ö., Bayrakdar İ.Ş., Orhan K., Bilgir E., Odabaş A., Aslan A.F. (2020). Success of Artificial Intelligence System in Determining Alveolar Bone Loss from Dental Panoramic Radiography Images. Cumhur. Dent. J..

[28] Cerda Mardini D., Cerda Mardini P., Vicuña Iturriaga D.P., Ortuño Borroto D.R. (2024). Determining the efficacy of a machine learning model for measuring periodontal bone loss. BMC Oral Health.

[29] Chang H.J., Lee S.J., Yong T.H., Shin N.Y., Jang B.G., Kim J.E., Huh K.H., Lee S.S., Heo M.S., Choi S.C. (2020). Deep Learning Hybrid Method to Automatically Diagnose Periodontal Bone Loss and Stage Periodontitis. Sci. Rep..

[30] Chang J., Chang M.F., Angelov N., Hsu C.Y., Meng H.W., Sheng S., Glick A., Chang K., He Y.-R., Lin Y.-B. (2022). Application of deep machine learning for the radiographic diagnosis of periodontitis. Clin. Oral Investig..

[31] Chen C.C., Wu Y.F., Aung L.M., Ngo S.T., Lin Y.-M., Chang W.-J. (2023). Automatic recognition of teeth and periodontal bone loss measurement in digital radiographs using deep-learning artificial intelligence. J. Dent. Sci..

[32] Dai F., Liu Q., Guo Y., Wu J., Zhu H., Xie R., Liu Q., Deng L., Song L. (2024). Convolutional neural networks combined with classification algorithms for the diagnosis of periodontitis. Oral Radiol..

[33] Danks R.P., Bano S., Orishko A., Tan H.J., Sancho F.M., Danks R.P., D’aIuto F. (2021). Automating Periodontal bone loss measurement via dental landmark localisation. Int. J. Comput. Assist. Radiol. Surg..

[34] Dujic H., Meyer O., Hoss P., Wölfle U.C., Wülk A., Meusburger T., Meier L., Gruhn V., Hesenius M., Hickel R. (2023). Automatized Detection of Periodontal Bone Loss on Periapical Radiographs by Vision Transformer Networks. Diagnostics.

[35] Guler Ayyildiz B., Karakis R., Terzioglu B., Ozdemir D. (2024). Comparison of deep learning methods for the radiographic detection of patients with different periodontitis stages. Dentomaxillofac. Radiol..

[36] Hoss P., Meyer O., Wölfle U.C., Wülk A., Meusburger T., Meier L., Hickel R., Gruhn V., Hesenius M., Hickel R. (2023). Detection of Periodontal Bone Loss on Periapical Radiographs—A Diagnostic Study Using Different Convolutional Neural Networks. J. Clin. Med..

[37] Jiang L., Chen D., Cao Z., Zhu F., Chen D., Zhu H. (2022). A two-stage deep learning architecture for radiographic staging of periodontal bone loss. BMC Oral Health.

[38] Jundaeng J., Chamchong R., Nithikathkul C. (2025). Advanced AI-assisted panoramic radiograph analysis for periodontal prognostication and alveolar bone loss detection. Front. Dent. Med..

[39] Kim J., Lee H.S., Song I.S., Jung K.H. (2019). DeNTNet: Deep Neural Transfer Network for the detection of periodontal bone loss using panoramic dental radiographs. Sci. Rep..

[40] Kong Z., Ouyang H., Cao Y., Huang T., Ahn E., Zhang M., Liu H. (2023). Automated periodontitis bone loss diagnosis in panoramic radiographs using a bespoke two-stage detector. Comput. Biol. Med..

[41] Moran M., Faria M., Giraldi G., Bastos L., Conci A. (2021). Do Radiographic Assessments of Periodontal Bone Loss Improve with Deep Learning Methods for Enhanced Image Resolution?. Sensors.

[42] Abu P.A.R., Mao Y.C., Lin Y.J., Chao C.-K., Lin Y.-H., Wang B.-S., Chen C.-A., Chen S.-L., Chen T.-Y., Li K.-C. (2025). Precision Medicine Assessment of the Radiographic Defect Angle of the Intrabony Defect in Periodontal Lesions by Deep Learning of Bitewing Radiographs. Bioengineering.

[43] Karacaoglu F., Kolsuz M.E., Bagis N., Evli C., Orhan K. (2023). Development and validation of intraoral periapical radiography-based machine learning model for periodontal defect diagnosis. Proc. Inst. Mech. Eng. H.

[44] Kurt-Bayrakdar S., Bayrakdar İ.Ş., Yavuz M.B., Sali N., Çelik Ö., Köse O., Saylan B.C.U., Kuleli B., Jagtap R., Orhan K. (2024). Detection of periodontal bone loss patterns and furcation defects from panoramic radiographs using deep learning algorithm: A retrospective study. BMC Oral Health.

[45] Mao Y.C., Huang Y.C., Chen T.Y., Chuo Y., Lin Y.-J., Chan M.-L., Chen C.-A., Li K.-C., Abu P.A.R., Huang Y.-C. (2023). Deep Learning for Dental Diagnosis: A Novel Approach to Furcation Involvement Detection on Periapical Radiographs. Bioengineering.

[46] Piroonsan K., Pimolbutr K., Tansriratanawong K. (2024). Classifying Three-Wall Intrabony Defects from Intraoral Radiographs Using Deep Learning-Based Convolutional Neural Network Models.. Eur. J. Dent..

[47] Shetty S., Talaat W., AlKawas S., Al-Rawi N., Reddy S., Hamdoon Z., Kheder W., Acharya A., Ozsahin D.U., David L.R. (2024). Application of artificial intelligence-based detection of furcation involvement in mandibular first molar using cone beam tomography images- a preliminary study. BMC Oral Health.

[48] Vilkomir K., Phen C., Baldwin F., Cole J., Herndon N., Zhang W. (2024). Classification of mandibular molar furcation involvement in periapical radiographs by deep learning. Imaging Sci. Dent..

[49] Zhang X., Guo E., Liu X., Li W., Zhao H., Yang J., Zhang X., Wu W. (2025). Enhancing furcation involvement classification on panoramic radiographs with vision transformers. BMC Oral Health.

[50] Ariandi V., Yanto M., Jamhur A.I., Firdaus F., Afira R. (2023). Optimization artificial neural network classification analysis model diagnosis Gingivitis disease. Indones. J. Electr. Eng. Comput. Sci..

[51] Alalharith D.M., Alharthi H.M., Alghamdi W.M., Alsenbel Y.M., Aslam N., Khan I.U., Shahin S.Y., Dianišková S., Alhareky M.S., Barouch K.K. (2020). A Deep Learning-Based Approach for the Detection of Early Signs of Gingivitis in Orthodontic Patients Using Faster Region-Based Convolutional Neural Networks. Int. J. Environ. Res. Public Health..

[52] Li W., Chen Y., Sun W., Brown M., Zhang X., Wang S., Miao L. (2019). A Gingivitis Identification Method Based on Contrast-Limited Adaptive Histogram Equalization, Gray-Level Co-Occurrence Matrix, and Extreme Learning Machine. Int. J. Imaging Syst. Technol..

[53] Li W., Guo E., Zhao H., Li Y., Miao L., Liu C., Sun W. (2024). Evaluation of Transfer Ensemble Learning-Based Convolutional Neural Network Models for the Identification of Chronic Gingivitis from Oral Photographs. BMC Oral Health..

[54] Li W., Liang Y., Zhang X., Liu C., He L., Miao L., Sun W. (2021). A Deep Learning Approach to Automatic Gingivitis Screening Based on Classification and Localization in RGB Photos. Sci. Rep..

[55] Wen C., Bai X., Yang J., Li S., Wang X., Yang D. (2024). Deep Learning-Based Approach: Automated Gingival Inflammation Grading Model Using Gingival Removal Strategy. Sci. Rep..

[56] Liu Y., Cheng Y., Song Y., Cai D., Zhang N. (2024). Oral Screening of Dental Calculus, Gingivitis, and Dental Caries Through Segmentation on Intraoral Photographic Images Using Deep Learning. BMC Oral Health..

[57] Chau R.C.W., Li G.H., Tew I.M., Thu K.M., McGrath C., Lo W.L., Ling W.K., Hsung R.T., Lam W.Y.H. (2023). Accuracy of Artificial Intelligence-Based Photographic Detection of Gingivitis. Int. Dent. J..

[58] Chau R.C.W., Cheng A.C.C., Mao K., Thu K.M., Ling Z., Tew I.M., Chang T.H., Tan H.J., McGrath C., Lo W.L. (2025). External Validation of an AI mHealth Tool for Gingivitis Detection Among Older Adults at Daycare Centers: A Pilot Study. Int. Dent. J..

[59] Andrade K.M., Silva B.P.M., de Oliveira L.R., Cury P.R. (2023). Automatic Dental Biofilm Detection Based on Deep Learning. J. Clin. Periodontol..

[60] Chen X., Shen Y., Jeong J.S., Perinpanayagam H., Kum K.Y., Gu Y. (2024). DeepPlaq: Dental Plaque Indexing Based on Deep Neural Networks. Clin. Oral Investig..

[61] Nantakeeratipat T., Apisaksirikul N., Boonrojsaree B., Boonkijkullatat S., Simaphichet A. (2024). Automated Machine Learning for Image-Based Detection of Dental Plaque on Permanent Teeth. Front. Dent. Med..

[62] Ramírez-Pedraza A., Salazar-Colores S., Cardenas-Valle C., Terven J., González-Barbosa J.J., Ornelas-Rodriguez F.J., Hurtado-Ramos J.B., Ramirez-Pedraza R., Córdova-Esparza D.M., Romero-González J.A. (2025). Deep Learning in Oral Hygiene: Automated Dental Plaque Detection via YOLO Frameworks and Quantification Using the O’Leary Index. Diagnostics.

[63] Wang C., Zhang R., Wei X., Wang L., Wu P., Yao Q. (2023). Deep Learning and Sub-Band Fluorescence Imaging-Based Method for Caries and Calculus Diagnosis Embeddable on Different Smartphones. Biomed. Opt. Express..

[64] Wang C., Zhang R., Wei X., Wang L., Xu W., Yao Q. (2023). Machine Learning-Based Automatic Identification and Diagnosis of Dental Caries and Calculus Using Hyperspectral Fluorescence Imaging. Photodiagnosis Photodyn. Ther..

[65] Li W., Li L., Xu W., Guo Y., Xu M., Huang S., Dai D., Lu C., Li S., Lin J. (2025). Identification of Gingival Inflammation Surface Image Features Using Intraoral Scanning and Deep Learning. Int. Dent. J..

[66] You W., Hao A., Li S., Wang Y., Xia B. (2020). Deep Learning-Based Dental Plaque Detection on Primary Teeth: A Comparison with Clinical Assessments. BMC Oral Health..

[67] Yüksel B., Özveren N., Yeşil Ç. (2024). Evaluation of Dental Plaque Area with Artificial Intelligence Model. Niger. J. Clin. Pract..

[68] Lin T.J., Lin Y.T., Lin Y.J., Tseng A.Y., Lin C.Y., Lo L.T., Chen T.Y., Chen S.L., Chen C.A., Li K.C. (2024). Auxiliary Diagnosis of Dental Calculus Based on Deep Learning and Image Enhancement by Bitewing Radiographs. Bioengineering.

[69] Orhan K., Aktuna Belgin C., Manulis D., Golitsyna M., Bayrak S., Aksoy S., Sanders A., Önder M., Ezhov M., Shamshiev M. (2023). Determining the Reliability of Diagnosis and Treatment Using Artificial Intelligence Software with Panoramic Radiographs. Imaging Sci. Dent..

[70] Shon H.S., Kong V., Park J.S., Jang W., Cha E.J., Kim S.Y., Lee E.Y., Kang T.G., Kim K.A. (2022). Deep Learning Model for Classifying Periodontitis Stages on Dental Panoramic Radiography. Appl. Sci..

[71] Ertaş K., Pence I., Cesmeli M.S., Ay Z.Y. (2023). Determination of the Stage and Grade of Periodontitis According to the Current Classification of Periodontal and Peri-Implant Diseases and Conditions (2018) Using Machine Learning Algorithms. J. Periodontal Implant Sci..

[72] Erturk M., Öziç M.Ü., Tassoker M. (2025). Deep Convolutional Neural Network for Automated Staging of Periodontal Bone Loss Severity on Bite-Wing Radiographs: An Eigen-CAM Explainability Mapping Approach. J. Imaging Inform. Med..

[73] Lee J.H., Kim D.H., Jeong S.N., Choi S.H. (2018). Diagnosis and Prediction of Periodontally Compromised Teeth Using a Deep Learning-Based Convolutional Neural Network Algorithm. J. Periodontal Implant Sci..

[74] Xue T., Chen L., Sun Q. (2024). Deep Learning Method to Automatically Diagnose Periodontal Bone Loss and Periodontitis Stage in Dental Panoramic Radiograph. J. Dent..

[75] Li X., Chen K., Zhao D., He Y., Li Y., Li Z., Guo X., Zhang C., Li W., Wang S. (2025). Deep Learning for Staging Periodontitis Using Panoramic Radiographs. Oral Dis..

[76] Machado V., Proença L., Morgado M., Mendes J.J., Botelho J. (2020). Accuracy of Panoramic Radiograph for Diagnosing Periodontitis Comparing to Clinical Examination. J. Clin. Med..

[77] Liu Y., Gao L., Jiang Y., Xu T., Peng L., Zhao X., Yang M., Li J., Liang S. (2025). AI-Aided Diagnosis of Periodontitis in Oral X-ray Images. Displays.

[78] Vigil M.S.A., Gowri V., Ramesh S.S.S., Praba M.S.B., Sabitha P. (2024). ADGRU: Adaptive DenseNet with Gated Recurrent Unit for Automatic Diagnosis of Periodontal Bone Loss and Stage Periodontitis with Tooth Segmentation Mechanism. Clin. Oral Investig..

[79] Ozden F.O., Özgönenel O., Özden B., Aydogdu A. (2015). Diagnosis of Periodontal Diseases Using Different Classification Algorithms: A Preliminary Study. Niger. J. Clin. Pract..

[80] Alam M.K., Alanazi N.H., Alshehri A.D.A., Chowdhury F. (2024). Accuracy of AI Algorithms in Diagnosing Periodontal Disease Using Intraoral Images. J. Pharm. Bioallied Sci..

[81] Aykol-Sahin G., Yucel O., Eraydin K., Aygün E., Dündar D. (2024). A Comparative Study of Neural Networks for Classification of Periodontal Disease Based on Radiographic Features. Oral Radiol..

[82] Gunpinar S., Sevinc A.S., Akgül Z., Tasmektepligil A.A., Gunpinar E. (2025). Patient-specific gingival recession system based on periodontal disease prediction. Int. J. Comput. Dent..

[83] Kurt-Bayrakdar S., Uğurlu M., Yavuz M.B., Sali N., Bayrakdar İ.Ş., Çelik Ö., Köse O., Beklen A., Uzun Saylan B.C., Jagtap R. (2023). Detection of tooth numbering, frenulum attachment, gingival overgrowth, and gingival inflammation signs on dental photographs using convolutional neural network algorithms: A retrospective study. Quintessence Int..

[84] Kearney V.P., Yansane A.M., Brandon R.G., Vaderhobli R., Lin G.H., Hekmatian H., Deng W., Joshi N., Bhandari H., Sadat A.S. (2022). A generative adversarial inpainting network to enhance prediction of periodontal clinical attachment level. J. Dent..

[85] Kim J.N. (2021). Investigation of the super-resolution algorithm for the prediction of periodontal disease in dental X-ray radiography. J. Korean Soc. Radiol..

[86] Motmaen I., Xie K., Schönbrunn L., Berens J., Grunert K., Plum A.M., Raufeisen J., Ferreira A., Hermans A., Egger J. (2024). Insights into predicting tooth extraction from panoramic dental images: Artificial intelligence vs. dentists, Clin. Oral Investig..

[87] Ossowska A., Kusiak A., Świetlik D. (2022). Evaluation of the progression of periodontitis with the use of neural networks. J. Clin. Med..

[88] Nguyen K.T., Le B.M., Li M., Almeida F.T., Major P.W., Kaipatur N.R., Lou E.H.M., Punithakumar K., Le L.H. (2021). Localization of cementoenamel junction in intraoral ultrasonographs with machine learning. J. Dent..

[89] Su S., Jia X., Zhan L., Gao S., Zhang Q., Huang X. (2024). Automatic tooth periodontal ligament segmentation of cone beam computed tomography based on instance segmentation network. Heliyon.

[90] You F.T., Lin P.C., Huang C.L., Wu J.H., Kabasawa Y., Chen C.C., Huang H.L. (2024). Artificial intelligence with counseling on the treatment outcomes and quality of life in periodontitis patients. J. Periodontol..

[91] Macrì M., D’Albis V., D’Albis G., Forte M., Capodiferro S., Favia G., Alrashadah A.O., García V.D., Festa F. (2024). The Role and Applications of Artificial Intelligence in Dental Implant Planning: A Systematic Review. Bioengineering.

[92] Mohammad-Rahimi H., Motamedian S.R., Pirayesh Z., Haiat A., Zahedrozegar S., Mahmoudinia E., Rohban M.H., Krois J., Lee J.H., Schwendicke F. (2022). Deep learning in periodontology and oral implantology: A scoping review. J. Periodontal Res..

[93] Mallineni S.K., Sethi M., Punugoti D., Kotha S.B., Alkhayal Z., Mubaraki S., Almotawah F.N., Kotha S.L., Sajja R., Nettam V. (2024). Artificial Intelligence in Dentistry: A Descriptive Review. Bioengineering.

[94] Ding H., Wu J., Zhao W., Matinlinna J.P., Burrow M.F., Tsoi J.K.H. (2023). Artificial intelligence in dentistry-A review. Front. Dent. Med..

